# Supplementary material for: Effect of a single dose of 8 mg moxidectin or 150 μg/kg ivermectin on O. volvulus skin microfilariae in a randomized trial: Differences between areas in the Democratic Republic of the Congo, Liberia and Ghana and impact of intensity of infection
Source: PLoS Negl Trop Dis. 2022 Apr 27;16(4):e0010079. doi: 10.1371/journal.pntd.0010079 (PMC9084535; doi:10.1371/journal.pntd.0010079)
Supplement: S1 File — Table S1 in S1 File: Sex, age and O. volvulus infection of individuals screened by study area and village. Fig S1 in S1 File: Distribution of skin microfilariae density among those screened by study area and sex. Fig S2 in S1 File: Skin microfilariae density across all individuals screened by study area and sex. Fig S3 in S1 File: Skin microfilariae density among those screened by study area, sex and age. Table S2 in S1 File: Community infection indicators among screened individuals ≥20 years by study area and village. Table S3 in S1 File: GPS coordinates of participant villages, research center and towns in the vicinity. Table S4 in S1 File: SmfD pre-treatment, 1, 6, 12 and 18 months post treatment across all study areas by intensity of infection pre-treatment. Table S5 in S1 File: SmfD pre-treatment, 1, 6, 12 and 18 months post treatment in Nord-Kivu (DRC) by intensity of infection pre-treatment. Table S6 in S1 File: SmfD pre-treatment, 1, 6, 12 and 18 months post treatment in Nord-Ituri (DRC) by intensity of infection pre-treatment. Table S7 in S1 File: SmfD pre-treatment, 1, 6, 12 and 18 months post treatment in Lofa County, Liberia, by intensity of infection pre-treatment. Table S8 in S1 File: SmfD pre-treatment, 1, 6, 12 and 18 months post treatment in Nkwanta district, Ghana, by intensity of infection pre-treatment. Table S9 in S1 File: Adjusted arithmetic and geometric means and mean differences in SmfD 1, 6, 12 and 18 months post treatment by study area. Table S10 in S1 File: Adjusted SmfD means and mean differences 12 months post-treatment by pre-treatment IoI. Table S11 in S1 File: Participants with undetectable SmfD from Month 1 to 6, 12 or 18 by IoI and area. Fig S4 in S1 File: Odds for UD from 1 to 6, 12 or 18 months by IoI and study area. Table S12 in S1 File: Logistic model derived odds for undetectable levels of skin microfilariae from month 1 sustained to month 6, month 12 or month 18 among participants treated with moxidectin or ivermectin by IoI [file pntd.0010079.s001.pdf]

## Supporting information S1_File:

### Screening data and analysis of skin microfilariae densities of study participants

Effect of a single dose of 8 mg moxidectin or 150 µg/kg ivermectin on *O. volvulus* skin microfilariae in a randomized trial: Differences between areas in the Democratic Republic of the Congo, Liberia and Ghana and impact of intensity of infection

Didier Bakajika[1,#a], Eric M Kanza[2,#b], Nicholas O Opoku[3,#c], Hayford M Howard[4,#d], Germain L Mambandu[1,#e], Amos Nyathirombo[1,#f], Maurice M Nigo[1,#g], Kambale Kasonia Kennedy[2, #h], Safari L Masembe[2,†],Mupenzi Mumbere[2], Kambale Kataliko[2,#i], Kpehe M Bolay[4,#j], Simon K Attah[3,#k], George Olipoh[3,#l], Sampson Asare[3,#m], Michel Vaillant[5], Christine M Halleux[6], Annette C Kuesel[6*]

[1] Centre de Recherche en Maladies Tropicale de l'Ituri, Hôpital Générale de Référence de Rethy, Ituri, Democratic Republic of the Congo (DRC)

[2] Centre de Recherche Clinique de Butembo, Université Catholique du Graben, Site Horizon, Butembo, Nord Kivu, DRC

[3] Onchocerciasis Chemotherapy Research Center, Hohoe, Ghana

[4] Clinical Research Center, Liberia Institute for Biomedical Research, Bolahun, Liberia

[5] Competence Center for Methodology and Statistics, Luxembourg Institute of Health, Strassen, Grand Duchy of Luxembourg

[6] UNICEF/UNDP/World Bank/WHO Special Programme for Research and Training in Tropical Diseases (WHO/TDR), World Health Organization, Geneva, Switzerland

[#a] Current Address: ESPEN, African Regional Office of the World Health Organization (WHO/AFRO/ESPEN), Brazzaville, Republic of Congo

[#b] Current Address: Programme Nationale de Lutte contre les Maladies Tropicales Négligées à Chimiothérapie Préventive (PNLMTN-CTP), Ministère de la Santé Publique, Kinshasa, Democratic Republic of the Congo (DRC)

[#c] Current Address: Department of Epidemiology and Biostatistics School of Public Health, University of Health and Allied Sciences, Hohoe, Ghana

[#d] Current Address: Ganta United Methodist Hospital, Ganta City, Nimba County, Liberia

[#e] Current Address: Inspection Provinciale de la Santé de la Tshopo, Division Provinciale de la Santé de la Tshopo, Kisangani, Province de la Tshopo, DRC

[#f] Current Address: Department of Ophthalmology, Faculty of Medicine, Gulu University, Uganda

[#g] Current Address: Institut Supérieur des Techniques Médicales de Nyankunde, Bunia, Ituri, DRC

[#h] Current Address: Department of Clinical Research, London School of Hygiene and Tropical Medicine, UK

[#i] Current Address: Centre de Santé CECA 20 de Mabakanga, Beni, Nord Kivu, DRC.

[#j] Current Address: National Public Health Institute of Liberia, Public Health & Medical Research, Monrovia, Liberia

[#k] Current Address: Department of Microbiology, University of Ghana Medical School, Accra, Ghana

[#l] Current Address: Precious Minerals Marketing Company Ltd., National Assay Centre, Technical Department, Diamond House, Accra, Ghana

[#m] Current Address: GlycoScience Research Inc, Brookings, South Dakota, United States of America

[†] Deceased

*Corresponding author: kuesela@who.int, ORCID ID: 0000-0002-1696-1784

# Table of Contents

Prevalence and intensity of *O. volvulus* infection among screened individuals by study area 3

Table S1 Sex, age and *O. volvulus* infection of individuals screened by study area and village 3

Fig S1 Distribution of skin microfilariae density among those screened by study area and sex 4

Fig S2 Skin microfilariae density across all individuals screened by study area and sex 5

Fig S3 Skin microfilariae density among those screened by study area, sex and age 6

Table S2: Community infection indicators among screened individuals ≥20 years by study area and village 7

Table S3: GPS coordinates of participant villages, research center and towns in the vicinity 8

Analysis of skin microfilariae densities of study participants 9

Table S4 SmfD pre-treatment, 1, 6, 12 and 18 months post treatment across all study areas by intensity of infection pre-treatment 9

Table S5 SmfD pre-treatment, 1, 6, 12 and 18 months post treatment in Nord-Kivu (DRC) by intensity of infection pre-treatment 10

Table S6 SmfD pre-treatment, 1, 6, 12 and 18 months post treatment in Nord-Ituri (DRC) by intensity of infection pre-treatment 11

Table S7 SmfD pre-treatment, 1, 6, 12 and 18 months post treatment in Lofa County, Liberia, by intensity of infection pre-treatment 12

Table S8 SmfD pre-treatment, 1, 6, 12 and 18 months post treatment in Nkwanta district, Ghana, by intensity of infection pre-treatment 13

Table S9 Adjusted arithmetic and geometric means and mean differences in SmfD 1, 6, 12 and 18 months post treatment by study area 14

Table S10 Adjusted SmfD means and mean differences 12 months post-treatment by pre-treatment IoI 15

Table S11 Participants with undetectable SmfD from Month 1 to 6, 12 or 18 by IoI and area 16

Fig S4: Odds for UD from 1 to 6, 12 or 18 months by IoI and study area 18

Table S12 Logistic model derived odds for undetectable levels of skin microfilariae from month 1 sustained to month 6, month 12 or month 18 among participants treated with moxidectin or ivermectin by IoI across and by study area 19

Table S13 Logistic model derived odds ratios for undetectable levels of skin microfilariae from month 1 sustained to month 6, month 12 or month 18 among participants treated with moxidectin relative to among ivermectin treated participants by IoI by study area 20

References 21

# Prevalence and intensity of *O. volvulus* infection among screened individuals by study area

Table S1 shows for each study area the number of individuals screened by sex, age group and whether skin microfilariae were detected (0 mf/mg skin vs. >0 mf/mg skin). Presentation by <20 and ≥20 years was chosen because evaluation of 30-50 men ≥ 20 years for infection as indicated by the presence of palpable subcutaneous nodules is the basis for rapid epidemiological assessment (REA) and rapid epidemiological mapping of onchocerciasis prevalence (REMO). REA and REMO were used to identify and map onchocerciasis endemicity in the APOC countries. Percentages of ≥20 year old individuals with palpable nodules of approximately 20-30% and above approximately 30%, respectively, indicate meso-endemic (infection prevalence ≈40-60 %) and hyperendemic areas (infection prevalence above ≈60%) [1–3]. In these areas CDTI was to be implemented to eliminate onchocerciasis as a public health problem [4–8] since infection prevalence >40% was found to be associated with an increased risk of onchocercal blindness [9].

Fig S1 , Fig S2 and Fig S3 provide overviews of SmfD among those screened by study area.

**Table S1 Sex, age and *O. volvulus* infection of individuals screened by study area and village**

| Category | Statistic/ parameter | DRC Nord Kivu | DRC Nord Ituri | Liberia Lofa | Ghana Nkwanta |
|---|---|---|---|---|---|
| Men | N | 733 | 885 | 581 | 384 |
| Women | N | 636 | 524 | 522 | 261 |
| | **Number screened (% within sex/age group indicated)** | | | | |
| Men <20 yrs | N | 9 (1.2%) | 109 (12.3%) | 24 (4.1%) | 108 (28.1%) |
| Men ≥20 yrs | N | 724 (98.8%) | 776 (87.7%) | 557 (95.9%) | 276 (71.9%) |
| Women <20 yrs | N | 17 (2.7%) | 47 (9.0%) | 4 (0.8%) | 93 (35.6%) |
| Women ≥20 yrs | N | 619 (97.3%) | 477 (91.0%) | 518 (99.2%) | 168 (64.4%) |
| | **Number by detectable SmfD (% within sex/age group)** | | | | |
| Men <20 yrs | 0 mf/mg skin | 8 (88.9%) | 39 (35.8%) | 15 (62.5%) | 15 (13.9%) |
| | >0 mf/mg skin | 1 (11.1%) | 70 (64.2%) | 9 (37.5%) | 93 (86.1%) |
| Men ≥20 yrs | 0 mf/mg skin | 200 (27.6%) | 251 (32.2%) | 246 (44.2%) | 30 (10.9%) |
| | >0 mf/mg skin | 524 (72.4%) | 525 (67.7%) | 311 (55.8%) | 246 (89.1%) |
| Women <20yrs | 0 mf/mg skin | 10 (58.8%) | 23 (48.9%) | 0 (0) | 21 (22.6%) |
| | >0 mf/mg skin | 7 (41.2%) | 24 (51.1%) | 4 (100%) | 72 (77.4%) |
| Women ≥20yrs | 0 mf/mg skin | 237 (38.3%) | 135 (28.3%) | 264 (51.0%) | 37 (22.0%) |
| | >0 mf/mg skin | 382 (61.7%) | 342 (71.7%) | 254 (49.0%) | 131 (78.0%) |
| | **Age** | | | | |
| Men <20 yrs | Mean±SD | 16.2±1.64 | 16.4±1.98 | 18.0±1.59 | 16.1±2.10 |
| | Min, Median, Max | 14, 16, 19 | 12, 17, 19 | 14, 19, 19 | 12, 16, 19 |
| Men ≥20 yrs | Mean±SD | 43.5±14.90 | 39.1±15.21 | 46.6±18.00 | 33.7±12.3 |
| | Min, Median, Max | 20, 43, 86 | 20, 36.5, 93 | 20, 45, 95 | 20, 30, 72 |
| Women <20yrs | Mean±SD | 16.5±1.91 | 15.7±2.00 | 17.0±2.71 | 15.7±2.30 |
| | Min, Median, Max | 13, 17, 19 | 12, 16, 19 | 13, 18, 19 | 12, 16, 19 |
| Women ≥20yrs | Mean±SD | 49.9±13.13 | 49.9±13.55 | 50.8±13.88 | 44.1±14.08 |
| | Min, Median, Max | 20, 50, 92 | 20, 50, 85 | 20, 50, 90 | 20, 44, 80 |

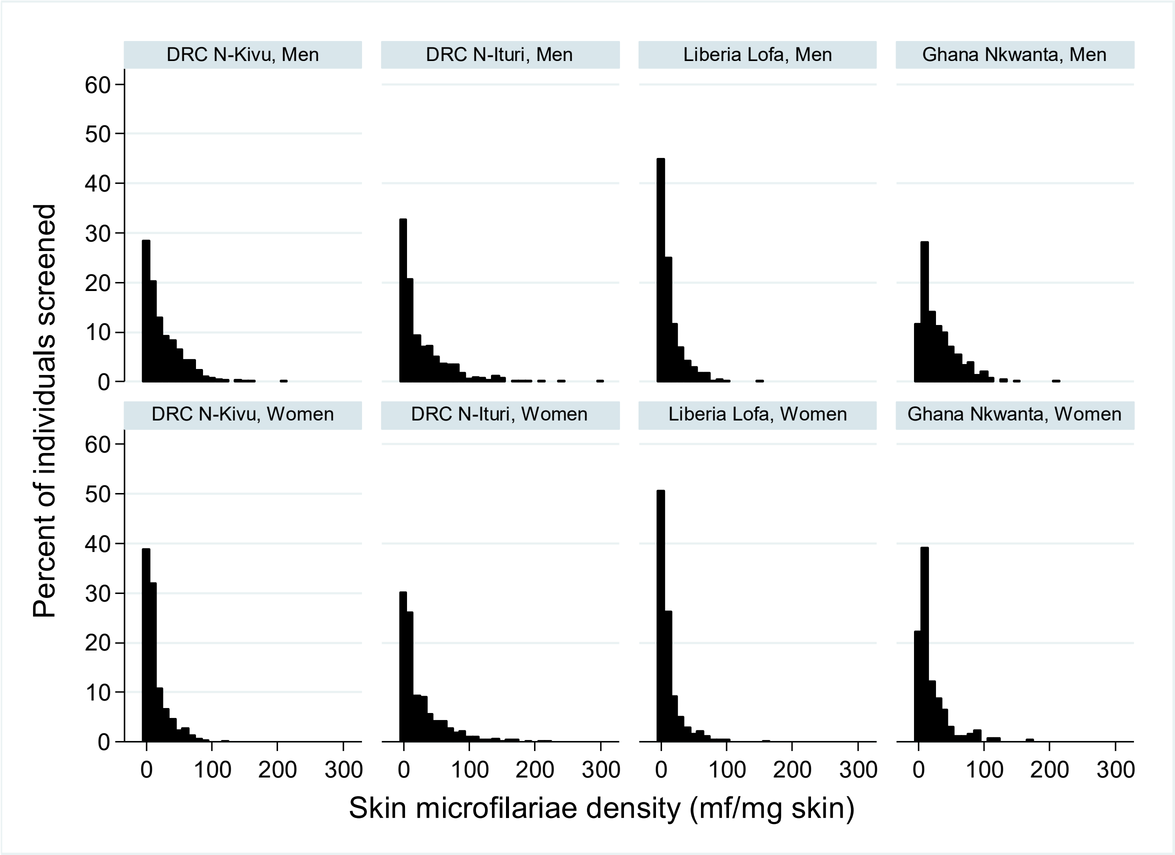


**Fig S1 Distribution of skin microfilariae density among those screened by study area and sex**

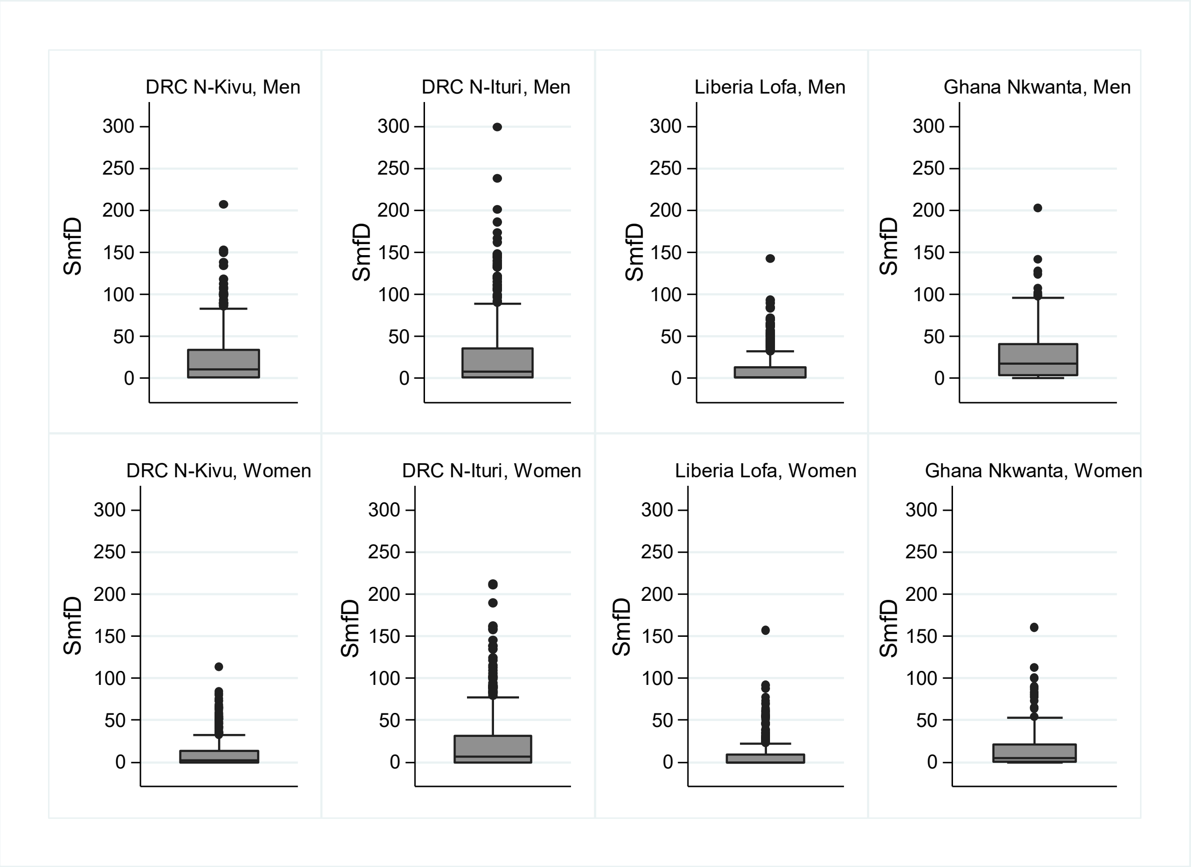


**Fig S2 Skin microfilariae density across all individuals screened by study area and sex**

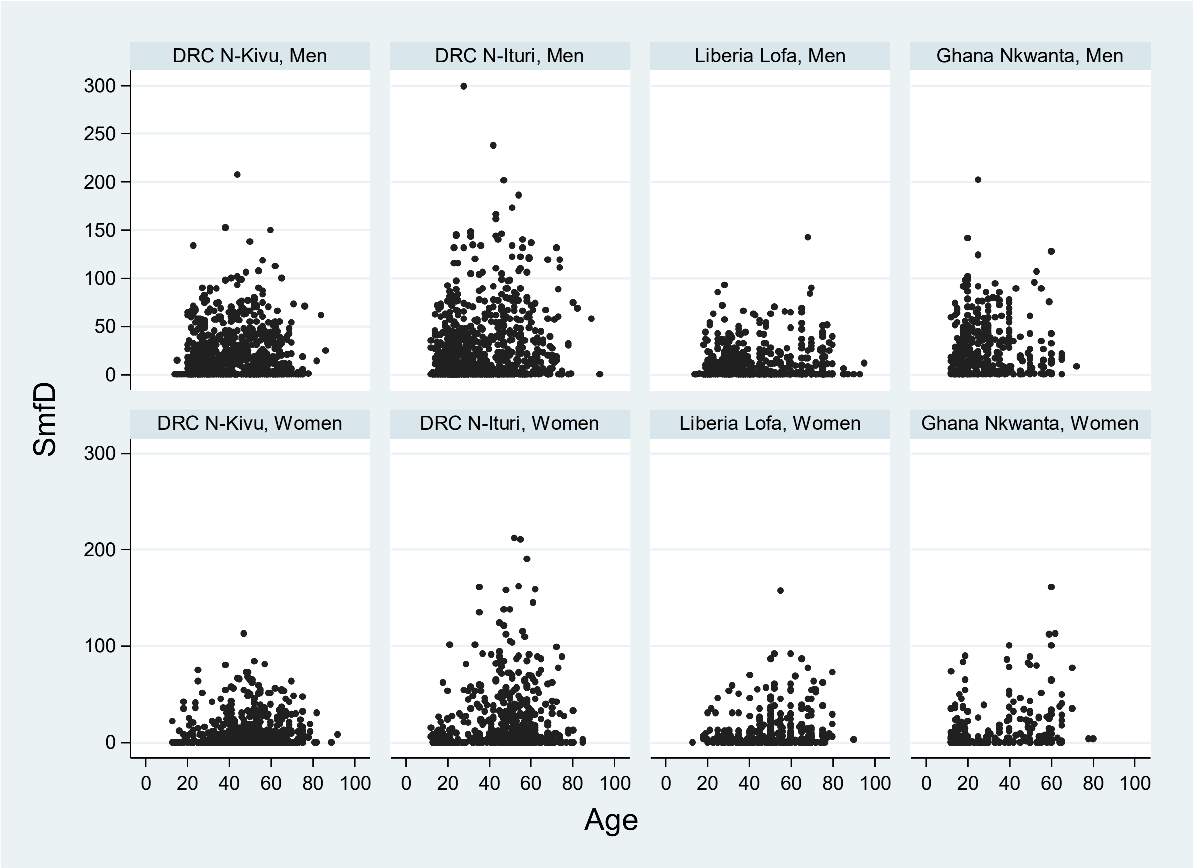


**Fig S3 Skin microfilariae density among those screened by study area, sex and age**

Table S2 shows for villages where at least 18 individuals were screened two indicators of onchocerciasis endemicity: the percentage of ≥20 year olds with detectable skin microfilariae levels and their screening microfilarial load (ScMFL), calculated in the same way as the Community Microfilarial Load (CMFL) [10]. GPS coordinates of the villages from where the majority of participants were recruited are provided in Table S3.

**Table S2: Community infection indicators among screened individuals ≥20 years by study area and village**

| Study area | Village | Number ≥20 years old screened | % ≥20 years old with detectable SmfD | ScMFL[1] | Number all ages enrolled (% enrolled) [2] |
|---|---|---|---|---|---|
| Nord Kivu | Kanyatsi | 26 | 30.8 | 1.0 | 2 (0.4%) |
| | Luala | 21 | 81.0 | 11.3 | 9 (2.0%) |
| | Mambingi | 479 | 70.4 | 5.9 | 178 (38.7%) |
| | Tutu | 21 | 71.4 | 4.3 | 6 (1.3%) |
| | Mambena | 164 | 76.8 | 7.7 | 74 (16.1) |
| | Masangi | 107 | 50.5 | 2.3 | 20 (4.3%) |
| | Visiki | 472 | 66.5 | 5.2 | 153 (33.3%) |
| Nord Ituri | Adrasi | 43 | 79.1 | 12.9 | 26 (5.5%) |
| | Draju | 48 | 70.8 | 9.4 | 22 (4.7%) |
| | Jupadrogo | 25 | 48.0 | 4.8 | 7 (1.5%) |
| | Jupagasa | 116 | 71.6 | 7.6 | 47 (10.0%) |
| | Kpana | 224 | 72.3 | 8.5 | 96 (20.3%) |
| | Ndroy | 67 | 89.6 | 24.0 | 37 (7.8%) |
| | Ngbungbu | 34 | 91.2 | 17.8 | 17 (3.6%) |
| | Rudju | 93 | 82.8 | 17.5 | 52 (11.0%) |
| | Umulo | 309 | 65.4 | 4.7 | 96 (20.3%) |
| | Yawu | 63 | 58.7 | 4.8 | 22 (4.7%) |
| | Juparima | 25 | 80.0 | 8.3 | 6 (1.3%) |
| | Kanga | 82 | 53.7 | 2.7 | 13 (2.8%) |
| | Ulyeko | 22 | 13.6 | 0.3 | 1 (0.2%) |
| Lofa | Fangoda | 88 | 19.3 | 0.6 | 9 (3.0%) |
| | Fokolahun | 79 | 72.2 | 6.9 | 36 (12.0%) |
| | Kamatahun | 138 | 68.1 | 4.9 | 63 (21.1%) |
| | Koilahun | 18 | 66.7 | 4.6 | 8 (2.7%) |
| | Massabolahun | 143 | 7.0 | 0.2 | 4 (1.3%) |
| | Mbalotahun | 20 | 25.0 | 1.9 | 2 (0.7%) |
| | Ngohombu | 24 | 95.8 | 9.9 | 12 (4.0%) |
| | Popalahun | 116 | 44.8 | 1.4 | 19 (6.4%) |
| | Porlowu | 107 | 28.0 | 0.6 | 8 (2.7%) |
| | Yallahun | 138 | 76.8 | 6.1 | 63 (21.1%) |
| | Yandohun | 52 | 73.1 | 7.9 | 25 (8.4%) |
| | Yengema | 152 | 79.6 | 4.8 | 50 (16.7%) |
| Nkwanta N | Azua | 277 | 80.1 | 6.9 | 154 (63.9%) |
| | Bitaaba | 74 | 94.6 | 19.8 | 22 (9.1%) |
| | Jagri Akura | 75 | 92.0 | 10.9 | 55 (22.8%) |
| | Wii | 18 | 88.9 | 14.7 | 10 (4.1%) |

[1]ScMFL – Microfilarial load for ≥20 year olds screened (Geometric mean (SmfD+1) – 1) for villages with ≥ 18 individuals ≥20 year olds screened. [2] % enrolled per study area, remaining participants were enrolled from villages in whom <18 individuals were screened.

**Table S3: GPS coordinates of participant villages, research center and towns in the vicinity**

| Study area | Village where participants lived | Research Center | Towns for reference | Latitude | Longitude |
|---|---|---|---|---|---|
| Nord Kivu, DRC | Mambena | | | 0.418306 | 29.225528 |
| Nord Kivu, DRC | Mambingi | | | 0.36656 | 29.27531 |
| Nord Kivu, DRC | Visiki | | | 0.37709 | 29.23835 |
| Nord Kivu, DRC | | Butembo | | 0.11405 | 29.30180 |
| Nord Kivu, DRC | | | Beni | 0.49901 | 29.45275 |
| Nord Ituri, DRC | Adrasi | | | 2.123217 | 30.969 |
| Nord Ituri, DRC | Draju | | | 2.118517 | 30.966067 |
| Nord Ituri, DRC | Jupadrogo | | | 2.1278 | 30.966067 |
| Nord Ituri, DRC | Jupagasa | | | 2.121 | 30.982133 |
| Nord Ituri, DRC | Juparima | | | 2.0868 | 30.955917 |
| Nord Ituri, DRC | Kanga | | | 2.074233 | 30.954283 |
| Nord Ituri, DRC | Kpana | | | 2.1114 | 30.96585 |
| Nord Ituri, DRC | Ndroy | | | 2.103917 | 30.974417 |
| Nord Ituri, DRC | Ngbungbu | | | 2.120117 | 30.97925 |
| Nord Ituri, DRC | Rudju | | | 2.124517 | 30.9835 |
| Nord Ituri, DRC | Ulyeko | | | 2.13545 | 30.956383 |
| Nord Ituri, DRC | Umulo | | | 2.131033 | 30.972117 |
| Nord Ituri, DRC | Yawu | | | 2.125017 | 30.965883 |
| Nord Ituri, DRC | | Rethy | | 2.07500 | 30.92165 |
| Nord Ituri, DRC | | | Bunia | 1.57428 | 30.23973 |
| Nord Ituri, DRC | | | Logo | 2.183167 | 30.927333 |
| Nord Ituri, DRC | | | Mahagi | 2.30547 | 30.97616 |
| Lofa County, Lib. | Kamatahun | | | 8.1368 | -10.297133 |
| Lofa County, Lib. | Yallahun | | | 8.131033 | -10.3124 |
| Lofa County, Lib. | Yandohun | | | 8.105333 | -10.35805 |
| Lofa County, Lib. | Yengma | | | 8.170283 | -10.307933 |
| Lofa County, Lib. | Fokolahun | | | 8.15935 | -10.274517 |
| Lofa County, Lib. | | Bolahun | | 8.23092 | -10.16064 |
| Lofa County, Lib. | | | Foya | 8.39492 | -10.21516 |
| Lofa County, Lib. | | | Lofa | 8.19112 | -9.72327 |
| Lofa County, Liberia | | | Voinjama | 8.42020 | -9.75393 |
| Nkwanta Dist., Ghana | Azua | | | 8.57208 | 0.40642 |
| Nkwanta Dist., Ghana | Bitaaba | | | 8.51159 | 0.31829 |
| Nkwanta Dist., Ghana | Jagri Akura | | | 8.53148 | 0.35278 |
| Nkwanta Dist., Ghana | Wii | | | 8.51974 | 0.33389 |
| Ghana | | Hohoe | | 7.15185 | 0.473829 |
| Nkwanta District, Ghana | | | Kpasa | 8.48962 | 0.30428 |
| Nkwanta District, Ghana | | | Nkwanta | 8.26031 | 0.52211 |
| Ghana | | | Wiae | 8.32275 | -0.16192 |
| Ghana | | | Asubende | 7.91667 | -1.16667 |
| Ghana | | | Kyingakrom | 8.10122 | -2.05871 |

# Analysis of skin microfilariae densities of study participants

**Table S4 SmfD pre-treatment, 1, 6, 12 and 18 months post treatment across all study areas by intensity of infection pre-treatment**

| Pre-Tx IoI (mf/mg) | Treatment Group | N | Raw Mean (SD) | Raw 95% CI | Raw Median (1Q,3Q) | Raw Min., Max. | Geometric Mean (95%CI) | Overall p-value (a) |
|---|---|---|---|---|---|---|---|---|
| Pre-treatment SmfD | | | | | | | | |
| 10 - <20 | Moxidectin | 281 | 13.9 (2.9) | [13.6,14.3] | 13.0 (11.0,16.0) | 9.0,19.0 | 13.7 [13.4,14.0] | <0.0001 |
| | Ivermectin | 150 | 14.1 (2.9) | [13.6,14.5] | 14.0 (11.0,17.0) | 10.0,19.0 | 13.8 [13.3,14.2] | |
| 20 - <50 | Moxidectin | 456 | 32.3 (8.6) | [31.5,33.1] | 31.0 (24.0,40.0) | 20.0,49.0 | 31.2 [30.4,32.0] | |
| | Ivermectin | 183 | 31.9 (8.3) | [30.7,33.1] | 31.0 (25.0,38.0) | 20.0,49.0 | 30.9 [29.7,32.1] | |
| 50 - <80 | Moxidectin | 157 | 62.4 (7.9) | [61.2,63.7] | 62.0 (56.0,69.0) | 50.0,79.0 | 61.9 [60.7,63.2] | |
| | Ivermectin | 108 | 62.0 (8.6) | [60.3,63.6] | 60.5 (55.0,69.0) | 50.0,79.0 | 61.4 [59.8,63.0] | |
| ≥80 | Moxidectin | 84 | 113.7 (37.0) | [105.6,121.7] | 100.5 (89.0,135.0) | 80.0,299.0 | 109.1 [102.8,115.8] | |
| | Ivermectin | 53 | 107.4 (32.7) | [98.4,116.5] | 93.0 (87.0,112.0) | 80.0,238.0 | 103.7 [96.7,111.3] | |
| Month 1 | | | | | | | | |
| 10 - <20 | Moxidectin | 281 | 0.0 (0.1) | [-0.0,0.0] | 0.0 (0.0,0.0) | 0.0,1.0 | 0.0 [-0.0,0.0] | <0.0001 |
| | Ivermectin | 150 | 0.9 (2.4) | [0.6,1.3] | 0.0 (0.0,1.0) | 0.0,16.0 | 0.4 [0.3,0.6] | |
| 20 - <50 | Moxidectin | 452 | 0.0 (0.2) | [0.0,0.0] | 0.0 (0.0,0.0) | 0.0,3.0 | 0.0 [0.0,0.0] | |
| | Ivermectin | 183 | 1.7 (5.4) | [0.9,2.5] | 0.0 (0.0,1.0) | 0.0,51.0 | 0.6 [0.4,0.8] | |
| 50 - <80 | Moxidectin | 157 | 0.1 (0.3) | [0.0,0.1] | 0.0 (0.0,0.0) | 0.0,3.0 | 0.0 [0.0,0.1] | |
| | Ivermectin | 106 | 3.2 (8.9) | [1.5,5.0] | 0.0 (0.0,2.0) | 0.0,54.0 | 0.9 [0.6,1.3] | |
| ≥80 | Moxidectin | 83 | 0.5 (1.3) | [0.2,0.8] | 0.0 (0.0,0.0) | 0.0,8.0 | 0.2 [0.1,0.4] | |
| | Ivermectin | 53 | 6.2 (13.8) | [2.4,10.0] | 0.0 (0.0,3.0) | 0.0,52.0 | 1.5 [0.8,2.5] | |
| Month 6 | | | | | | | | |
| 10 - <20 | Moxidectin | 275 | 0.0 (0.1) | [-0.0,0.0] | 0.0 (0.0,0.0) | 0.0,2.0 | 0.0 [-0.0,0.0] | <0.0001 |
| | Ivermectin | 150 | 1.3 (2.6) | [0.9,1.8] | 1.0 (0.0,1.0) | 0.0,21.0 | 0.8 [0.6,1.0] | |
| 20 - <50 | Moxidectin | 449 | 0.0 (0.1) | [0.0,0.0] | 0.0 (0.0,0.0) | 0.0,1.0 | 0.0 [0.0,0.0] | |
| | Ivermectin | 181 | 2.6 (4.4) | [2.0,3.3] | 1.0 (0.0,3.0) | 0.0,31.0 | 1.5 [1.2,1.8] | |
| 50 - <80 | Moxidectin | 154 | 0.0 (0.1) | [-0.0,0.0] | 0.0 (0.0,0.0) | 0.0,1.0 | 0.0 [-0.0,0.0] | |
| | Ivermectin | 107 | 5.1 (7.2) | [3.8,6.5] | 2.0 (1.0,7.0) | 0.0,36.0 | 2.8 [2.1,3.5] | |
| ≥80 | Moxidectin | 84 | 0.0 (0.2) | [0.0,0.1] | 0.0 (0.0,0.0) | 0.0,1.0 | 0.0 [0.0,0.1] | |
| | Ivermectin | 53 | 10.5 (10.2) | [7.7,13.3] | 7.0 (3.0,17.0) | 0.0,43.0 | 6.5 [4.7,8.9] | |
| Month 12 | | | | | | | | |
| 10 - <20 | Moxidectin | 270 | 0.4 (1.0) | [0.2,0.5] | 0.0 (0.0,0.0) | 0.0,8.0 | 0.2 [0.1,0.3] | <0.0001 |
| | Ivermectin | 148 | 3.4 (6.0) | [2.5,4.4] | 2.0 (1.0,4.0) | 0.0,58.0 | 1.9 [1.6,2.4] | |
| 20 - <50 | Moxidectin | 440 | 1.1 (2.7) | [0.9,1.4] | 0.0 (0.0,1.0) | 0.0,18.0 | 0.5 [0.4,0.6] | |
| | Ivermectin | 177 | 7.0 (8.3) | [5.7,8.2] | 4.0 (2.0,10.0) | 0.0,49.0 | 4.2 [3.5,4.9] | |
| 50 - <80 | Moxidectin | 154 | 2.3 (4.4) | [1.6,3.0] | 0.0 (0.0,2.0) | 0.0,28.0 | 1.0 [0.7,1.3] | |
| | Ivermectin | 103 | 15.2 (13.0) | [12.6,17.7] | 11.0 (6.0,22.0) | 0.0,49.0 | 10.0 [8.0,12.3] | |
| ≥80 | Moxidectin | 83 | 2.6 (4.5) | [1.6,3.5] | 1.0 (0.0,2.0) | 0.0,20.0 | 1.2 [0.8,1.6] | |
| | Ivermectin | 52 | 28.6 (18.1) | [23.5,33.6] | 24.5 (15.0,38.5) | 1.0,77.0 | 22.5 [17.9,28.1] | |
| Month 18 | | | | | | | | |
| 10 - <20 | Moxidectin | 270 | 0.4 (1.0) | [0.2,0.5] | 0.0 (0.0,0.0) | 0.0,8.0 | 0.2 [0.1,0.3] | <0.0001 |
| | Ivermectin | 148 | 3.4 (6.0) | [2.5,4.4] | 2.0 (1.0,4.0) | 0.0,58.0 | 1.9 [1.6,2.4] | |
| 20 - <50 | Moxidectin | 440 | 1.1 (2.7) | [0.9,1.4] | 0.0 (0.0,1.0) | 0.0,18.0 | 0.5 [0.4,0.6] | |
| | Ivermectin | 177 | 7.0 (8.3) | [5.7,8.2] | 4.0 (2.0,10.0) | 0.0,49.0 | 4.2 [3.5,4.9] | |
| 50 - <80 | Moxidectin | 154 | 2.3 (4.4) | [1.6,3.0] | 0.0 (0.0,2.0) | 0.0,28.0 | 1.0 [0.7,1.3] | |
| | Ivermectin | 103 | 15.2 (13.0) | [12.6,17.7] | 11.0 (6.0,22.0) | 0.0,49.0 | 10.0 [8.0,12.3] | |
| ≥80 | Moxidectin | 83 | 2.6 (4.5) | [1.6,3.5] | 1.0 (0.0,2.0) | 0.0,20.0 | 1.2 [0.8,1.6] | |
| | Ivermectin | 52 | 28.6 (18.1) | [23.5,33.6] | 24.5 (15.0,38.5) | 1.0,77.0 | 22.5 [17.9,28.1] | |

(a) p-value for the difference in SmfD at the indicated month between IoI categories, obtained from linear model where mean skin microfilarial density at 1, 6, 12 or 18 months was the outcome variable with treatment as explanatory variable controlling for other covariates such as intensity of infection pre-treatment and sex.
p = <0.0001 for IoI <20, 20-<50, 50-<80, ≥80 for the interaction of IoI category and treatment across SmfD at Month 1, 6, 12, 18, obtained from linear model where mean skin microfilarial density at 1, 6, 12 or 18 months was the outcome variable with treatment as explanatory variable controlling for other covariates such as intensity of infection pre-treatment and sex.
SmfD were logarithmic transformed (y'=log(y+1)) before analysis and back transformed to give geometric means and difference of geometric means in the dimension of the raw data.

**Table S5 SmfD pre-treatment, 1, 6, 12 and 18 months post treatment in Nord-Kivu (DRC) by intensity of infection pre-treatment**

| Pre-Tx IoI (mf/mg) | Treatment Group | N | Raw Mean (SD) | Raw 95% CI | Raw Median (1Q,3Q) | Raw Min., Max. | Geometric Mean [95%CI] | Overall p-value (a) |
|---|---|---|---|---|---|---|---|---|
| Pre-treatment SmfD | | | | | | | | |
| 10 - <20 | Moxidectin | 92 | 13.7 (3.0) | [13.0,14.3] | 13.2 (11.0,16.2) | 9.7,19.5 | 13.4 [12.8,14.0] | <0.0001 |
| | Ivermectin | 51 | 14.2 (3.3) | [13.3,15.2] | 14.7 (10.9,17.1) | 9.7,19.3 | 13.9 [13.0,14.8] | |
| 20 - <50 | Moxidectin | 151 | 31.8 (8.7) | [30.4,33.2] | 31.3 (23.8,39.7) | 19.5,49.5 | 30.7 [29.3,32.1] | |
| | Ivermectin | 53 | 32.4 (8.3) | [30.1,34.6] | 30.8 (25.4,37.5) | 20.7,48.5 | 31.4 [29.2,33.6] | |
| ≥50 | Moxidectin | 62 | 71.5 (21.5) | [66.0,76.9] | 65.4 (56.4,75.4) | 51.2,150.0 | 69.0 [64.7,73.6] | |
| | Ivermectin | 51 | 70.8 (27.9) | [63.0,78.7] | 61.3 (55.5,74.1) | 49.6,207.2 | 67.4 [62.1,73.2] | |
| Month 1 | | | | | | | | |
| 10 - <20 | Moxidectin | 92 | 0.0 (0.1) | [-0.0,0.0] | 0.0 (0.0,0.0) | 0.0,1.0 | 0.0 [-0.0,0.0] | <0.0001 |
| | Ivermectin | 51 | 0.5 (0.9) | [0.3,0.8] | 0.2 (0.0,0.6) | 0.0,3.8 | 0.4 [0.2,0.6] | |
| 20 - <50 | Moxidectin | 148 | 0.0 (0.1) | [0.0,0.1] | 0.0 (0.0,0.0) | 0.0,0.8 | 0.0 [0.0,0.0] | |
| | Ivermectin | 53 | 1.8 (3.9) | [0.7,2.9] | 0.3 (0.0,0.9) | 0.0,18.2 | 0.8 [0.4,1.2] | |
| ≥50 | Moxidectin | 62 | 0.1 (0.3) | [0.0,0.2] | 0.0 (0.0,0.0) | 0.0,1.5 | 0.1 [0.0,0.1] | |
| | Ivermectin | 49 | 4.3 (8.7) | [1.8,6.8] | 0.7 (0.0,3.1) | 0.0,44.8 | 1.6 [0.9,2.5] | |
| Month 6 | | | | | | | | |
| 10 - <20 | Moxidectin | 89 | 0.0 (0.2) | [-0.0,0.1] | 0.0 (0.0,0.0) | 0.0,1.7 | 0.0 [-0.0,0.0] | <0.0001 |
| | Ivermectin | 51 | 0.9 (1.3) | [0.5,1.2] | 0.4 (0.1,1.2) | 0.0,7.4 | 0.6 [0.4,0.9] | |
| 20 - <50 | Moxidectin | 148 | 0.0 (0.0) | [0.0,0.0] | 0.0 (0.0,0.0) | 0.0,0.5 | 0.0 [0.0,0.0] | |
| | Ivermectin | 51 | 2.6 (4.9) | [1.3,4.0] | 0.9 (0.3,2.9) | 0.0,28.0 | 1.4 [0.9,2.0] | |
| ≥50 | Moxidectin | 61 | 0.0 (0.1) | [-0.0,0.1] | 0.0 (0.0,0.0) | 0.0,0.7 | 0.0 [0.0,0.1] | |
| | Ivermectin | 50 | 6.8 (9.3) | [4.2,9.5] | 2.9 (0.9,8.6) | 0.0,36.3 | 3.5 [2.4,5.0] | |
| Month 12 | | | | | | | | |
| 10 - <20 | Moxidectin | 90 | 0.1 (0.2) | [0.0,0.1] | 0.0 (0.0,0.0) | 0.0,1.3 | 0.1 [0.0,0.1] | <0.0001 |
| | Ivermectin | 51 | 1.6 (1.7) | [1.1,2.1] | 0.9 (0.4,2.3) | 0.0,7.1 | 1.2 [0.8,1.6] | |
| 20 - <50 | Moxidectin | 147 | 0.4 (0.8) | [0.3,0.6] | 0.0 (0.0,0.5) | 0.0,4.9 | 0.3 [0.2,0.4] | |
| | Ivermectin | 50 | 6.3 (9.6) | [3.6,9.0] | 2.7 (1.2,5.7) | 0.0,49.3 | 3.3 [2.2,4.6] | |
| ≥50 | Moxidectin | 61 | 1.1 (2.4) | [0.5,1.7] | 0.2 (0.0,0.8) | 0.0,12.1 | 0.6 [0.3,0.8] | |
| | Ivermectin | 48 | 16.9 (15.5) | [12.4,21.4] | 14.7 (3.4,23.3) | 0.0,70.3 | 10.2 [7.1,14.4] | |
| Month 18 | | | | | | | | |
| 10 - <20 | Moxidectin | 61 | 0.5 (1.4) | [0.2,0.9] | 0.0 (0.0,0.2) | 0.0,9.0 | 0.3 [0.1,0.4] | <0.0001 |
| | Ivermectin | 34 | 2.3 (2.6) | [1.4,3.2] | 1.6 (0.4,2.7) | 0.0,12.1 | 1.6 [1.1,2.3] | |
| 20 - <50 | Moxidectin | 87 | 1.4 (2.2) | [0.9,1.9] | 0.2 (0.0,2.3) | 0.0,10.7 | 0.8 [0.6,1.1] | |
| | Ivermectin | 34 | 7.9 (10.2) | [4.3,11.5] | 3.5 (0.4,15.2) | 0.0,49.8 | 3.8 [2.2,6.2] | |
| ≥50 | Moxidectin | 53 | 3.4 (6.0) | [1.8,5.1] | 1.2 (0.0,3.3) | 0.0,26.5 | 1.6 [1.0,2.3] | |
| | Ivermectin | 33 | 22.6 (18.5) | [16.0,29.1] | 19.6 (7.0,35.9) | 0.0,63.3 | 13.7 [8.8,21.1] | |

(a) p-value obtained from linear model where mean skin microfilarial density at 1, 6, 12 or 18 months was the outcome variable with treatment as explanatory variable controlling for other covariates such as intensity of infection pre-treatment and sex.

p = <0.0001 for IoI <20, 20-<50, ≥80 for the interaction of IoI category and treatment across SmfD at Month 1, 6, 12, 18, obtained from linear model where mean skin microfilarial density at 1, 6, 12 or 18 months was the outcome variable with treatment as explanatory variable controlling for other covariates such as intensity of infection pre-treatment and sex.

SmfD were logarithmic transformed (y'=log (y+1)) before analysis and back transformed to give geometric means and difference of geometric means in the dimension of the raw data.

**Table S6 SmfD pre-treatment, 1, 6, 12 and 18 months post treatment in Nord-Ituri (DRC) by intensity of infection pre-treatment**

| Pre-Tx IoI (mf/mg) | Treatment Group | N | Raw Mean (SD) | Raw 95% CI | Raw Median (1Q,3Q) | Raw Min., Max. | Geometric Mean (95%CI) | Overall p-value (a) |
|---|---|---|---|---|---|---|---|---|
| Pre-treatment SmfD | | | | | | | | |
| 10 - <20 | Moxidectin | 64 | 13.9 (2.9) | [13.2,14.6] | 13.9 (11.6,16.3) | 9.6,19.5 | 13.6 [12.9,14.4] | <0.0001 |
| | Ivermectin | 33 | 14.1 (2.9) | [13.1,15.1] | 13.2 (12.0,16.4) | 9.9,19.4 | 13.8 [12.8,14.9] | |
| 20 - <50 | Moxidectin | 145 | 32.9 (8.5) | [31.5,34.3] | 31.9 (25.5,40.2) | 19.8,49.3 | 31.8 [30.4,33.2] | |
| | Ivermectin | 53 | 32.5 (8.8) | [30.0,34.9] | 32.3 (23.7,39.9) | 20.2,49.4 | 31.3 [29.0,33.8] | |
| ≥50 | Moxidectin | 106 | 89.6 (41.6) | [81.6,97.6] | 73.3 (61.3,104.0) | 49.6,299.4 | 82.7 [76.9,89.0] | |
| | Ivermectin | 71 | 85.5 (33.3) | [77.6,93.4] | 77.7 (60.7,97.4) | 50.2,238.3 | 80.7 [74.6,87.2] | |
| Month 1 | | | | | | | | |
| 10 - <20 | Moxidectin | 64 | 0.0 (0.1) | [-0.0,0.0] | 0.0 (0.0,0.0) | 0.0,0.6 | 0.0 [-0.0,0.0] | 0.014 |
| | Ivermectin | 33 | 1.6 (3.2) | [0.5,2.8] | 0.2 (0.0,1.1) | 0.0,11.5 | 0.8 [0.3,1.3] | |
| 20 - <50 | Moxidectin | 145 | 0.1 (0.4) | [0.0,0.2] | 0.0 (0.0,0.1) | 0.0,3.4 | 0.1 [0.0,0.1] | |
| | Ivermectin | 53 | 2.1 (5.8) | [0.5,3.7] | 0.1 (0.0,1.5) | 0.0,34.6 | 0.8 [0.4,1.2] | |
| ≥50 | Moxidectin | 105 | 0.4 (1.2) | [0.2,0.7] | 0.0 (0.0,0.3) | 0.0,8.4 | 0.2 [0.1,0.4] | |
| | Ivermectin | 71 | 4.7 (12.5) | [1.7,7.6] | 0.2 (0.0,1.7) | 0.0,51.9 | 1.1 [0.6,1.7] | |
| Month 6 | | | | | | | | |
| 10 - <20 | Moxidectin | 64 | 0.0 (0.0) | [-0.0,0.0] | 0.0 (0.0,0.0) | 0.0,0.1 | 0.0 [-0.0,0.0] | <0.0001 |
| | Ivermectin | 33 | 1.9 (4.0) | [0.5,3.3] | 0.5 (0.0,1.3) | 0.0,21.2 | 0.9 [0.5,1.6] | |
| 20 - <50 | Moxidectin | 142 | 0.0 (0.1) | [0.0,0.0] | 0.0 (0.0,0.0) | 0.0,0.4 | 0.0 [0.0,0.0] | |
| | Ivermectin | 53 | 2.9 (4.1) | [1.8,4.1] | 1.6 (0.3,3.9) | 0.0,21.6 | 1.8 [1.2,2.5] | |
| ≥50 | Moxidectin | 106 | 0.0 (0.1) | [0.0,0.1] | 0.0 (0.0,0.0) | 0.0,1.2 | 0.0 [0.0,0.1] | |
| | Ivermectin | 71 | 8.2 (9.2) | [6.1,10.4] | 4.3 (1.7,11.7) | 0.0,43.1 | 4.8 [3.6,6.4] | |
| Month 12 | | | | | | | | |
| 10 - <20 | Moxidectin | 62 | 0.4 (0.7) | [0.2,0.6] | 0.0 (0.0,0.4) | 0.0,3.6 | 0.3 [0.2,0.4] | <0.0001 |
| | Ivermectin | 33 | 5.9 (10.8) | [2.1,9.7] | 2.3 (0.5,6.5) | 0.0,57.5 | 2.7 [1.5,4.4] | |
| 20 - <50 | Moxidectin | 140 | 1.2 (2.5) | [0.8,1.7] | 0.2 (0.0,0.9) | 0.0,14.9 | 0.6 [0.5,0.8] | |
| | Ivermectin | 53 | 8.5 (7.9) | [6.4,10.7] | 7.1 (3.0,10.2) | 0.0,35.5 | 5.8 [4.3,7.7] | |
| ≥50 | Moxidectin | 106 | 2.4 (3.9) | [1.6,3.1] | 0.7 (0.1,3.0) | 0.0,20.1 | 1.2 [0.9,1.6] | |
| | Ivermectin | 70 | 22.3 (17.0) | [18.3,26.4] | 17.3 (9.3,34.7) | 0.0,76.5 | 16.1 [12.8,20.1] | |
| Month 18 | | | | | | | | |
| 10 - <20 | Moxidectin | 59 | 2.0 (2.5) | [1.3,2.6] | 0.7 (0.1,3.0) | 0.0,10.3 | 1.2 [0.8,1.7] | <0.0001 |
| | Ivermectin | 28 | 8.5 (10.2) | [4.5,12.5] | 5.5 (2.1,9.3) | 0.0,41.2 | 5.1 [3.2,7.8] | |
| 20 - <50 | Moxidectin | 125 | 4.9 (9.1) | [3.3,6.5] | 1.6 (0.3,5.3) | 0.0,63.2 | 2.2 [1.7,2.8] | |
| | Ivermectin | 46 | 14.5 (13.5) | [10.5,18.5] | 9.8 (7.0,18.7) | 0.1,57.4 | 9.8 [7.3,13.2] | |
| ≥50 | Moxidectin | 99 | 11.3 (14.4) | [8.4,14.1] | 5.5 (0.9,18.4) | 0.0,70.5 | 5.3 [4.0,7.0] | |
| | Ivermectin | 68 | 35.1 (24.3) | [29.2,41.0] | 30.1 (15.8,50.1) | 0.5,101.3 | 26.4 [21.3,32.6] | |

(a) p-value obtained from linear model where mean skin microfilarial density at 1, 6, 12 or 18 months was the outcome variable with treatment as explaining variable controlling for other covariates such as intensity of infection pre-treatment and sex.

p = <0.0001 for IoI <20, 20-<50, ≥80 for the interaction of IoI category and treatment across SmfD at Month 1, 6, 12, 18, obtained from linear model where mean skin microfilarial density at 1, 6, 12 or 18 months was the outcome variable with treatment as explanatory variable controlling for other covariates such as intensity of infection pre-treatment and sex.

SmfD were logarithmic transformed (y'=log(y+1)) before analysis and back transformed to give geometric means and difference of geometric means in the dimension of the raw data.

**Table S7 SmfD pre-treatment, 1, 6, 12 and 18 months post treatment in Lofa County, Liberia, by intensity of infection pre-treatment**

| Pre-Tx IoI (mf/mg) | Treatment Group | N | Raw Mean (SD) | Raw 95% CI | Raw Median (1Q,3Q) | Raw Min., Max. | Geometric Mean (95%CI) | Overall p-value (a) |
|---|---|---|---|---|---|---|---|---|
| Pre-treatment SmfD | | | | | | | | |
| 10 - <20 | Moxidectin | 83 | 13.9 (2.8) | [13.3,14.5] | 13.1 (11.8,16.4) | 9.2,19.4 | 13.7 [13.1,14.3] | <0.0001 |
| | Ivermectin | 42 | 13.5 (2.7) | [12.7,14.3] | 13.3 (10.9,15.3) | 9.7,18.8 | 13.3 [12.5,14.1] | |
| 20 - <50 | Moxidectin | 84 | 31.9 (7.8) | [30.2,33.6] | 30.7 (24.9,38.5) | 19.5,47.3 | 31.0 [29.4,32.7] | |
| | Ivermectin | 39 | 30.2 (7.7) | [27.7,32.7] | 28.0 (23.9,33.4) | 19.7,48.6 | 29.4 [27.1,31.8] | |
| ≥50 | Moxidectin | 33 | 70.1 (24.2) | [61.5,78.7] | 63.4 (54.9,73.8) | 50.3,157.0 | 67.2 [60.9,74.1] | |
| | Ivermectin | 18 | 64.1 (12.2) | [58.0,70.2] | 61.3 (54.6,69.1) | 51.2,90.4 | 63.1 [57.8,69.0] | |
| Month 1 | | | | | | | | |
| 10 - <20 | Moxidectin | 83 | 0.0 (0.1) | [-0.0,0.0] | 0.0 (0.0,0.0) | 0.0,0.5 | 0.0 [-0.0,0.0] | 0.053 |
| | Ivermectin | 42 | 0.7 (1.8) | [0.1,1.3] | 0.0 (0.0,0.4) | 0.0,9.8 | 0.3 [0.1,0.6] | |
| 20 - <50 | Moxidectin | 84 | 0.0 (0.1) | [0.0,0.0] | 0.0 (0.0,0.0) | 0.0,0.4 | 0.0 [0.0,0.0] | |
| | Ivermectin | 39 | 0.7 (1.3) | [0.2,1.1] | 0.0 (0.0,0.8) | 0.0,6.0 | 0.4 [0.2,0.7] | |
| ≥50 | Moxidectin | 33 | 0.1 (0.2) | [-0.0,0.1] | 0.0 (0.0,0.0) | 0.0,1.0 | 0.0 [0.0,0.1] | |
| | Ivermectin | 18 | 4.3 (12.6) | [-2.0,10.5] | 0.1 (0.0,1.7) | 0.0,53.6 | 1.0 [0.1,2.5] | |
| Month 6 | | | | | | | | |
| 10 - <20 | Moxidectin | 80 | 0.0 (0.0) | [-0.0,0.0] | 0.0 (0.0,0.0) | 0.0,0.3 | 0.0 [-0.0,0.0] | <0.0001 |
| | Ivermectin | 42 | 0.9 (1.0) | [0.6,1.2] | 0.6 (0.2,1.2) | 0.0,4.0 | 0.7 [0.5,1.0] | |
| 20 - <50 | Moxidectin | 83 | 0.0 (0.2) | [-0.0,0.1] | 0.0 (0.0,0.0) | 0.0,1.1 | 0.0 [-0.0,0.0] | |
| | Ivermectin | 39 | 1.6 (2.6) | [0.8,2.5] | 0.9 (0.3,1.7) | 0.0,15.3 | 1.1 [0.7,1.5] | |
| ≥50 | Moxidectin | 33 | 0.1 (0.2) | [-0.0,0.1] | 0.0 (0.0,0.0) | 0.0,1.4 | 0.0 [-0.0,0.1] | |
| | Ivermectin | 18 | 3.9 (5.2) | [1.3,6.5] | 1.9 (0.5,5.2) | 0.0,21.5 | 2.3 [1.1,4.1] | |
| Month 12 | | | | | | | | |
| 10 - <20 | Moxidectin | 77 | 0.5 (1.3) | [0.2,0.8] | 0.0 (0.0,0.2) | 0.0,8.3 | 0.2 [0.1,0.4] | <0.0001 |
| | Ivermectin | 41 | 2.4 (2.7) | [1.6,3.3] | 1.4 (0.8,2.9) | 0.0,10.3 | 1.7 [1.1,2.3] | |
| 20 - <50 | Moxidectin | 78 | 1.1 (2.9) | [0.5,1.8] | 0.1 (0.0,0.8) | 0.0,18.2 | 0.5 [0.3,0.8] | |
| | Ivermectin | 36 | 4.7 (6.2) | [2.6,6.8] | 3.0 (1.1,5.4) | 0.0,34.0 | 3.0 [2.0,4.2] | |
| ≥50 | Moxidectin | 32 | 1.8 (3.8) | [0.5,3.2] | 0.2 (0.0,1.5) | 0.0,15.8 | 0.8 [0.3,1.4] | |
| | Ivermectin | 16 | 12.0 (11.1) | [6.1,17.9] | 7.5 (5.4,17.6) | 0.6,43.8 | 8.5 [5.0,13.9] | |
| Month 18 | | | | | | | | |
| 10 - <20 | Moxidectin | 75 | 1.1 (2.2) | [0.6,1.6] | 0.2 (0.0,1.0) | 0.0,14.9 | 0.6 [0.4,0.9] | <0.0001 |
| | Ivermectin | 40 | 3.5 (3.3) | [2.4,4.5] | 2.1 (1.0,5.1) | 0.0,13.0 | 2.5 [1.7,3.4] | |
| 20 - <50 | Moxidectin | 75 | 2.0 (3.7) | [1.2,2.9] | 0.3 (0.0,2.0) | 0.0,16.0 | 0.9 [0.6,1.4] | |
| | Ivermectin | 37 | 7.2 (9.3) | [4.1,10.3] | 3.4 (2.2,7.7) | 0.0,40.9 | 3.9 [2.5,6.0] | |
| ≥50 | Moxidectin | 33 | 4.6 (7.1) | [2.1,7.1] | 1.6 (0.0,5.7) | 0.0,27.1 | 2.0 [1.0,3.4] | |
| | Ivermectin | 16 | 15.6 (16.5) | [6.8,24.4] | 11.5 (3.9,22.7) | 1.7,66.4 | 10.1 [5.7,17.3] | |

(a) p-value obtained from linear model where mean skin microfilarial density at 1, 6, 12 or 18 months was the outcome variable with treatment as explaining variable controlling for other covariates such as intensity of infection pre-treatment and sex.

p = <0.0001 for IoI <20, 20-<50, ≥80 for the interaction of IoI category and treatment across SmfD at Month 1, 6, 12, 18, obtained from linear model where mean skin microfilarial density at 1, 6, 12 or 18 months was the outcome variable with treatment as explanatory variable controlling for other covariates such as intensity of infection pre-treatment and sex.

SmfD were logarithmic transformed (y'=log(y+1)) before analysis and back transformed to give geometric means and difference of geometric means in the dimension of the raw data.

**Table S8 SmfD pre-treatment, 1, 6, 12 and 18 months post treatment in Nkwanta district, Ghana, by intensity of infection pre-treatment**

| Pre-Tx IoI | Treatment Group | N | Raw Mean (SD) | Raw 95% CI | Raw Median (1Q,3Q) | Raw Min., Max. | Geometric Mean (95%CI) | Overall p-value (a) |
|---|---|---|---|---|---|---|---|---|
| Pre-treatment SmfD | | | | | | | | |
| 10 - <20 | Moxidectin | 42 | 14.7 (2.8) | [13.9,15.6] | 14.5 (12.3,17.6) | 10.5,19.0 | 14.5 [13.6,15.4] | <0.0001 |
| | Ivermectin | 24 | 14.5 (2.6) | [13.4,15.6] | 14.5 (13.1,17.1) | 10.0,18.6 | 14.3 [13.2,15.5] | |
| 20 - <50 | Moxidectin | 76 | 32.5 (9.5) | [30.3,34.6] | 31.9 (23.2,40.5) | 19.6,49.2 | 31.2 [29.1,33.3] | |
| | Ivermectin | 38 | 32.1 (8.3) | [29.4,34.8] | 32.1 (24.6,37.8) | 20.4,48.1 | 31.1 [28.5,33.9] | |
| ≥50 | Moxidectin | 40 | 77.8 (23.3) | [70.4,85.3] | 75.0 (60.5,90.7) | 49.8,160.6 | 75.0 [68.7,81.8] | |
| | Ivermectin | 21 | 73.8 (19.4) | [65.0,82.6] | 71.4 (58.5,82.4) | 49.6,124.0 | 71.6 [63.9,80.2] | |
| Month 1 | | | | | | | | |
| 10 - <20 | Moxidectin | 42 | 0.0 (0.0) | [-0.0,0.0] | 0.0 (0.0,0.0) | 0.0,0.2 | 0.0 [-0.0,0.0] | 0.448 |
| | Ivermectin | 24 | 1.5 (3.7) | [-0.0,3.1] | 0.2 (0.0,0.6) | 0.0,15.9 | 0.6 [0.2,1.3] | |
| 20 - <50 | Moxidectin | 75 | 0.0 (0.0) | [0.0,0.0] | 0.0 (0.0,0.0) | 0.0,0.2 | 0.0 [0.0,0.0] | |
| | Ivermectin | 38 | 2.3 (8.5) | [-0.5,5.1] | 0.0 (0.0,0.5) | 0.0,51.3 | 0.6 [0.2,1.1] | |
| ≥50 | Moxidectin | 40 | 0.1 (0.2) | [0.0,0.1] | 0.0 (0.0,0.1) | 0.0,1.1 | 0.1 [0.0,0.1] | |
| | Ivermectin | 21 | 2.9 (7.7) | [-0.6,6.4] | 0.2 (0.0,2.2) | 0.0,34.8 | 0.9 [0.2,2.0] | |
| Month 6 | | | | | | | | |
| 10 - <20 | Moxidectin | 42 | 0.0 (0.1) | [0.0,0.0] | 0.0 (0.0,0.0) | 0.0,0.3 | 0.0 [0.0,0.0] | 0.105 |
| | Ivermectin | 24 | 2.5 (3.8) | [0.9,4.1] | 1.1 (0.5,3.0) | 0.2,17.1 | 1.6 [0.9,2.5] | |
| 20 - <50 | Moxidectin | 76 | 0.0 (0.1) | [0.0,0.1] | 0.0 (0.0,0.0) | 0.0,0.9 | 0.0 [0.0,0.1] | |
| | Ivermectin | 38 | 3.4 (5.2) | [1.7,5.1] | 1.8 (0.8,4.8) | 0.0,31.0 | 2.1 [1.4,3.0] | |
| ≥50 | Moxidectin | 38 | 0.0 (0.1) | [0.0,0.1] | 0.0 (0.0,0.0) | 0.0,0.4 | 0.0 [0.0,0.1] | |
| | Ivermectin | 21 | 5.2 (7.0) | [2.1,8.4] | 2.1 (1.6,6.1) | 0.2,29.7 | 3.2 [1.8,5.2] | |
| Month 12 | | | | | | | | |
| 10 - <20 | Moxidectin | 41 | 0.8 (1.6) | [0.3,1.3] | 0.1 (0.0,0.8) | 0.0,8.3 | 0.5 [0.3,0.8] | <0.0001 |
| | Ivermectin | 23 | 5.5 (5.2) | [3.2,7.7] | 4.4 (1.6,6.5) | 0.0,22.9 | 3.9 [2.4,5.9] | |
| 20 - <50 | Moxidectin | 75 | 2.5 (4.2) | [1.6,3.5] | 0.6 (0.0,3.4) | 0.0,18.4 | 1.3 [0.8,1.8] | |
| | Ivermectin | 38 | 7.8 (8.4) | [5.0,10.6] | 5.6 (2.3,11.3) | 0.0,40.3 | 4.9 [3.4,7.1] | |
| ≥50 | Moxidectin | 38 | 5.2 (6.8) | [2.9,7.4] | 2.2 (0.1,6.2) | 0.0,27.9 | 2.5 [1.4,4.0] | |
| | Ivermectin | 21 | 23.0 (15.7) | [15.8,30.1] | 18.0 (8.1,34.2) | 0.5,49.2 | 17.1 [11.2,26.0] | |
| Month 18 | | | | | | | | |
| 10 - <20 | Moxidectin | 32 | 2.8 (4.2) | [1.3,4.4] | 1.0 (0.0,3.2) | 0.0,18.9 | 1.5 [0.8,2.4] | <0.0001 |
| | Ivermectin | 15 | 9.6 (7.5) | [5.5,13.8] | 8.2 (3.5,13.1) | 0.0,29.8 | 7.0 [4.0,11.9] | |
| 20 - <50 | Moxidectin | 49 | 7.9 (11.1) | [4.7,11.1] | 3.1 (1.5,8.2) | 0.0,47.5 | 4.0 [2.7,5.7] | |
| | Ivermectin | 23 | 14.6 (10.1) | [10.2,18.9] | 14.1 (6.2,20.2) | 0.2,41.7 | 10.9 [7.3,16.2] | |
| ≥50 | Moxidectin | 16 | 13.6 (19.2) | [3.4,23.8] | 3.3 (0.9,25.1) | 0.0,70.0 | 5.2 [1.9,12.2] | |
| | Ivermectin | 12 | 33.4 (16.5) | [22.9,43.9] | 32.1 (22.9,42.2) | 4.7,61.7 | 28.8 [18.8,43.7] | |

(a) p-value for the difference in SmfD at the indicated month between IoI categories, obtained from linear model where mean skin microfilarial density at 1, 6, 12 or 18 months was the outcome variable with treatment as explanatory variable controlling for other covariates such as intensity of infection pre-treatment and sex.
p = <0.0001 for IoI <20, 20-<50, ≥50 for the interaction of IoI category and treatment across SmfD at Month 1, 6, 12, 18, obtained from linear model where mean skin microfilarial density at 1, 6, 12 or 18 months was the outcome variable with treatment as explanatory variable controlling for other covariates such as intensity of infection pre-treatment and sex.
SmfD were logarithmic transformed (y'=log(y+1)) before analysis and back transformed to give geometric means and difference of geometric means in the dimension of the raw data.

**Table S9 Adjusted arithmetic and geometric means and mean differences in SmfD 1, 6, 12 and 18 months post treatment by study area**

| Study area<br>Time post treatment | Treatment Group | Adjusted Mean (95%CI) | Difference of adj. means (95%CI) | Adjusted Geometric Mean (95%CI) | Difference of Adj geometric Mean (95%CI) | Percent Diffe-rence | p-value° |
|---|---|---|---|---|---|---|---|
| **Nord Kivu** | | | | | | | |
| Month 1 | Moxidectin | 0.0 [-0.1;0.1] | | 0.0 [-0.1;0.1] | | | |
| | Ivermectin | 0.6 [0.5;0.7] | 0.5 [0.4;0.7] | 0.7 [0.6;0.9] | 0.7 (0.6;0.8) | 98.57 | <0.0001 |
| Month 6 | Moxidectin | -0.0 [-0.1;0.1] | | -0.0 [-0.1;0.1] | | | |
| | Ivermectin | 0.9 [0.8;1.0] | 0.9 [0.8;1.0] | 1.4 [1.2;1.7] | 1.4 (1.3;1.6) | 100.42 | <0.0001 |
| Month 12 | Moxidectin | 0.2 [0.1;0.3] | | 0.2 [0.2;0.3] | | | |
| | Ivermectin | 1.5 [1.4;1.6] | 1.3 [1.1;1.4] | 3.4 [3.0;3.9] | 3.2 (2.8;3.5) | 92.90 | <0.0001 |
| Month 18 | Moxidectin | 0.6 [0.5;0.6] | | 0.8 [0.6;0.9] | | | |
| | Ivermectin | 1.8 [1.6;1.9] | 1.2 [1.1;1.3] | 4.8 [4.2;5.4] | 4.0 (3.5;4.5) | 84.12 | <0.0001 |
| **Nord Ituri** | | | | | | | |
| Month 1 | Moxidectin | 0.1 [0.0;0.2] | | 0.1 [0.0;0.2] | | | |
| | Ivermectin | 0.6 [0.4;0.7] | 0.5 [0.3;0.6] | 0.8 [0.6;1.0] | 0.6 (0.5;0.8) | 83.70 | <0.0001 |
| Month 6 | Moxidectin | 0.0 [-0.1;0.1] | | 0.0 [-0.1;0.1] | | | |
| | Ivermectin | 1.2 [1.1;1.3] | 1.2 [1.0;1.3] | 2.4 [2.0;2.8] | 2.3 (2.0;2.7) | 98.76 | <0.0001 |
| Month 12 | Moxidectin | 0.6 [0.5;0.6] | | 0.7 [0.6;0.9] | | | |
| | Ivermectin | 2.1 [2.0;2.3] | 1.6 [1.4;1.7] | 7.5 [6.5;8.7] | 6.8 (5.9;7.7) | 90.14 | <0.0001 |
| Month 18 | Moxidectin | 1.3 [1.2;1.4] | | 2.8 [2.5;3.2] | | | |
| | Ivermectin | 2.6 [2.5;2.8] | 1.3 [1.1;1.4] | 12.8 [11.1;14.8] | 10.0 (8.6;11.6) | 77.97 | <0.0001 |
| **Lofa County** | | | | | | | |
| Month 1 | Moxidectin | 0.0 [-0.1;0.1] | | 0.0 [-0.1;0.1] | | | |
| | Ivermectin | 0.5 [0.4;0.6] | 0.5 [0.3;0.6] | 0.6 [0.4;0.9] | 0.6 (0.5;0.7) | 97.07 | <0.0001 |
| Month 6 | Moxidectin | 0.0 [-0.1;0.1] | | 0.0 [-0.1;0.1] | | | |
| | Ivermectin | 0.8 [0.7;1.0] | 0.8 [0.7;1.0] | 1.3 [1.0;1.6] | 1.3 (1.1;1.5) | 98.75 | <0.0001 |
| Month 12 | Moxidectin | 0.4 [0.3;0.5] | | 0.4 [0.3;0.6] | | | |
| | Ivermectin | 1.5 [1.3;1.6] | 1.1 [0.9;1.3] | 3.3 [2.8;3.9] | 2.9 (2.5;3.3) | 86.75 | <0.0001 |
| Month 18 | Moxidectin | 0.7 [0.6;0.8] | | 1.0 [0.8;1.2] | | | |
| | Ivermectin | 1.7 [1.6;1.8] | 1.0 [0.9;1.2] | 4.4 [3.8;5.2] | 3.5 (3.0;4.0) | 78.23 | <0.0001 |
| **Nkwanta N** | | | | | | | |
| Month 1 | Moxidectin | -0.1 [-0.2;0.0] | | -0.1 [-0.2;0.0] | | | |
| | Ivermectin | 0.5 [0.4;0.7] | 0.6 [0.4;0.8] | 0.7 [0.4;1.0] | 0.8 (0.6;1.0) | 110.69 | <0.0001 |
| Month 6 | Moxidectin | -0.1 [-0.2;0.1] | | -0.1 [-0.2;0.1] | | | |
| | Ivermectin | 1.2 [1.0;1.3] | 1.2 [1.0;1.5] | 2.2 [1.7;2.8] | 2.3 (1.9;2.8) | 103.21 | <0.0001 |
| Month 12 | Moxidectin | 0.7 [0.6;0.8] | | 1.0 [0.8;1.3] | | | |
| | Ivermectin | 2.0 [1.9;2.2] | 1.3 [1.1;1.5] | 6.6 [5.4;8.0] | 5.6 (4.6;6.7) | 84.48 | <0.0001 |
| Month 18 | Moxidectin | 1.3 [1.2;1.5] | | 2.9 [2.3;3.5] | | | |
| | Ivermectin | 2.6 [2.4;2.8] | 1.3 [1.0;1.5] | 13.1 [10.5;16.2] | 10.2 (8.2;12.8) | 78.15 | <0.0001 |

p-value for the difference in SmfD after moxidectin and ivermectin at each follow up time point obtained by marginal (least square means) means pairwise comparisons from the mixed effect model per study area where microfilarial density at 1, 6, 12 and 18 months was the outcome variable with treatment, follow up time point (1, 6, 12, 18 months), and interaction between treatment and follow up time point and interaction between treatment and intensity of infection at baseline as fixed effect, controlling for other covariates such as baseline density, baseline intensity of infection and sex.

>100% difference due to low SmfD values in the moxidectin treatment arm that are log-transformed to calculate the geometric means.

**Table S10 Adjusted SmfD means and mean differences 12 months post-treatment by pre-treatment IoI**

| Study area Screening intensity of Infection | Treatment Group | Adjusted Mean (95%CI) | Difference of adj. means (95%CI) | Adjusted Geometric Mean (95%CI) | Difference of Adj geometric Mean (95%CI) | Percent Difference | p-value° |
|---|---|---|---|---|---|---|---|
| **Across all** | | | | | | | |
| 10 - <20 mf/mg | Moxidectin | 0.5 [0.3;0.7] | | 0.6 [0.3;0.9] | | | |
| | Ivermectin | 1.2 [1.0;1.4] | 0.7 [0.6;0.8] | 2.3 [1.7;3.0] | 1.7 (1.4;2.1) | 74.10 | <0.0001 |
| 20 - <50 mf/mg | Moxidectin | 0.3 [0.2;0.5] | | 0.4 [0.2;0.6] | | | |
| | Ivermectin | 1.3 [1.1;1.4] | 0.9 [0.8;1.0] | 2.6 [2.0;3.2] | 2.2 (1.8;2.6) | 84.21 | <0.0001 |
| 50 - <80 mf/mg | Moxidectin | 0.3 [0.1;0.5] | | 0.4 [0.1;0.7] | | | |
| | Ivermectin | 1.5 [1.4;1.7] | 1.2 [1.1;1.4] | 3.7 [2.9;4.7] | 3.3 (2.7;4.1) | 89.98 | <0.0001 |
| ≥80 mf/mg | Moxidectin | 0.2 [-0.0;0.5] | | 0.3 [0.0;0.6] | | | |
| | Ivermectin | 1.9 [1.7;2.2] | 1.7 [1.5;1.9] | 5.7 [4.2;7.6] | 5.5 (4.2;7.0) | 95.38 | <0.0001 |
| **Nord Kivu** | | | | | | | |
| 10 - <20 mf/mg | Moxidectin | 0.5 [0.3;0.6] | | 0.6 [0.3;0.9] | | | |
| | Ivermectin | 1.0 [0.8;1.2] | 0.6 [0.4;0.7] | 1.8 [1.3;2.4] | 1.2 (1.0;1.5) | 68.15 | <0.0001 |
| 20 - <50 mf/mg | Moxidectin | 0.2 [0.1;0.3] | | 0.2 [0.1;0.3] | | | |
| | Ivermectin | 1.1 [1.0;1.2] | 0.9 [0.7;1.1] | 2.0 [1.6;2.4] | 1.8 (1.5;2.1) | 89.62 | <0.0001 |
| ≥50 mf/mg | Moxidectin | -0.1 [-0.2;0.1] | | 0.0 [-0.2;0.2] | | | |
| | Ivermectin | 1.4 [1.2;1.6] | 1.4 [1.2;1.6] | 3.0 [2.2;3.9] | 3.0 (2.5;3.8) | 101.63 | <0.0001 |
| **Nord Ituri** | | | | | | | |
| 10 - <20 mf/mg | Moxidectin | 0.6 [0.4;0.9] | | 0.9 [0.5;1.4] | | | |
| | Ivermectin | 1.6 [1.3;1.8] | 0.9 [0.7;1.2] | 3.7 [2.6;5.2] | 2.8 (2.1;3.8) | 75.73 | <0.0001 |
| 20 - <50 mf/mg | Moxidectin | 0.5 [0.4;0.6] | | 0.6 [0.5;0.8] | | | |
| | Ivermectin | 1.6 [1.4;1.7] | 1.1 [0.9;1.3] | 3.8 [3.1;4.7] | 3.2 (2.7;3.9) | 83.90 | <0.0001 |
| ≥50 mf/mg | Moxidectin | 0.4 [0.2;0.6] | | 0.5 [0.3;0.8] | | | |
| | Ivermectin | 1.8 [1.6;2.0] | 1.4 [1.2;1.6] | 4.9 [3.9;6.2] | 4.4 (3.7;5.4) | 89.91 | <0.0001 |
| **Lofa County** | | | | | | | |
| 10 - <20 mf/mg | Moxidectin | 0.2 [0.1;0.4] | | 0.2 [0.1;0.5] | | | |
| | Ivermectin | 0.8 [0.6;1.0] | 0.6 [0.5;0.8] | 1.3 [0.9;1.8] | 1.1 (0.9;1.3) | 81.16 | <0.0001 |
| 20 - <50 mf/mg | Moxidectin | 0.2 [0.1;0.3] | | 0.3 [0.1;0.4] | | | |
| | Ivermectin | 1.0 [0.9;1.1] | 0.8 [0.6;0.9] | 1.7 [1.3;2.1] | 1.5 (1.2;1.7) | 85.29 | <0.0001 |
| ≥50 mf/mg | Moxidectin | 0.4 [0.1;0.6] | | 0.4 [0.1;0.9] | | | |
| | Ivermectin | 1.5 [1.2;1.8] | 1.2 [0.9;1.4] | 3.6 [2.4;5.1] | 3.1 (2.3;4.2) | 87.85 | <0.0001 |
| **Nkwanta** | | | | | | | |
| 10 - <20 mf/mg | Moxidectin | 0.4 [0.1;0.7] | | 0.5 [0.2;1.0] | | | |
| | Ivermectin | 1.4 [1.1;1.8] | 1.0 [0.7;1.3] | 3.2 [2.1;4.8] | 2.7 (1.9;3.8) | 83.87 | <0.0001 |
| 20 - <50 mf/mg | Moxidectin | 0.5 [0.4;0.6] | | 0.6 [0.4;0.9] | | | |
| | Ivermectin | 1.4 [1.3;1.6] | 1.0 [0.7;1.2] | 3.3 [2.5;4.2] | 2.6 (2.1;3.3) | 80.18 | <0.0001 |
| ≥50 mf/mg | Moxidectin | 0.5 [0.2;0.8] | | 0.7 [0.2;1.3] | | | |
| | Ivermectin | 1.9 [1.6;2.2] | 1.4 [1.1;1.7] | 5.7 [3.8;8.4] | 5.0 (3.5;7.1) | 88.22 | <0.0001 |

p-value for the difference in SmfD after moxidectin and ivermectin for each IoI category obtained by marginal (least square means) means pairwise comparisons from the mixed effect model across and per study area where microfilarial density at 12 months was the outcome variable with treatment, baseline intensity of infection and sex as covariates

**Table S11 Participants with undetectable SmfD from Month 1 to 6, 12 or 18 by IoI and area**

| Area | Moxidectin | | | | | Ivermectin | | | | |
|---|---|---|---|---|---|---|---|---|---|---|
| **IoI** | **ND** | **n UD Yes** | **n UD No** | **N** | **% with UD** | **ND** | **n UD Yes** | **n UD No** | **N** | **% with UD** |
| **All areas** | | | | | | | | | | |
| UD 1-6 | | | | | | | | | | |
| Any | 20 | 741 | 217 | 958 | 77.3 | 5 | 35 | 454 | 489 | 7.2 |
| 10-<20 | 6 | 245 | 30 | 275 | 89.1 | | 18 | 132 | 150 | 12 |
| 20-<50 | 10 | 354 | 92 | 446 | 79.4 | 2 | 14 | 167 | 181 | 7.7 |
| 50-<80 | 3 | 107 | 47 | 154 | 69.5 | 3 | 3 | 102 | 105 | 2.9 |
| ≥80 | 1 | 35 | 48 | 83 | 42.2 | | 0 | 53 | 53 | 0 |
| UD 1-12 | | | | | | | | | | |
| Any | 40 | 360 | 578 | 938 | 38.4 | 16 | 7 | 471 | 478 | 1.5 |
| <20 | 14 | 159 | 108 | 267 | 59.6 | 2 | 3 | 145 | 148 | 2 |
| 20-<50 | 20 | 156 | 280 | 436 | 35.8 | 6 | 3 | 174 | 177 | 1.7 |
| 50-<80 | 4 | 32 | 121 | 153 | 20.9 | 7 | 1 | 100 | 101 | 1 |
| 80+ | 2 | 13 | 69 | 82 | 15.9 | 1 | 0 | 52 | 52 | 0 |
| UD 1-18 | | | | | | | | | | |
| Any | 222 | 164 | 592 | 756 | 21.7 | 111 | 4 | 379 | 383 | 1 |
| <20 | 55 | 76 | 150 | 226 | 33.6 | 33 | 1 | 116 | 117 | 0.9 |
| 20-<50 | 124 | 68 | 264 | 332 | 20.5 | 44 | 2 | 137 | 139 | 1.4 |
| 50-<80 | 27 | 15 | 115 | 130 | 11.5 | 25 | 1 | 82 | 83 | 1.2 |
| 80+ | 16 | 5 | 63 | 68 | 7.4 | 9 | 0 | 44 | 44 | 0 |
| | | | | | | | | | | |
| **N Kivu** | | | | | | | | | | |
| UD 1-6 | | | | | | | | | | |
| Any | 9 | 246 | 50 | 296 | 83.1 | 3 | 17 | 133 | 150 | 11.3 |
| 10-<20 | 3 | 80 | 9 | 89 | 89.9 | | 7 | 44 | 51 | 13.7 |
| 20-<50 | 5 | 123 | 23 | 146 | 84.2 | 2 | 8 | 43 | 51 | 15.7 |
| ≥50 | 1 | 43 | 18 | 61 | 70.5 | 1 | 2 | 46 | 48 | 4.2 |
| UD 1-12 | | | | | | | | | | |
| Any | 14 | 145 | 146 | 291 | 49.8 | 9 | 4 | 143 | 147 | 2.7 |
| <20 | 5 | 60 | 27 | 87 | 69 | | 2 | 49 | 51 | 3.9 |
| 20-<50 | 7 | 67 | 77 | 144 | 46.5 | 4 | 1 | 49 | 50 | 2 |
| ≥50 | 2 | 18 | 42 | 60 | 30 | 5 | 1 | 45 | 46 | 2.2 |
| UD 1-18 | | | | | | | | | | |
| Any | 106 | 73 | 126 | 199 | 36.7 | 55 | 3 | 97 | 100 | 3 |
| <20 | 31 | 31 | 30 | 61 | 50.8 | 17 | 1 | 33 | 34 | 2.9 |
| 20-<50 | 65 | 32 | 54 | 86 | 37.2 | 19 | 1 | 33 | 34 | 2.9 |
| ≥50 | 10 | 10 | 42 | 52 | 19.2 | 19 | 1 | 31 | 32 | 3.1 |
| | | | | | | | | | | |
| **N Ituri** | | | | | | | | | | |
| UD 1-6 | | | | | | | | | | |
| Any | 4 | 205 | 106 | 311 | 65.9 | 0 | 9 | 148 | 157 | 5.7 |
| <20 | 0 | 56 | 8 | 64 | 87.5 | | 5 | 28 | 33 | 15.2 |
| 20-<50 | 3 | 96 | 46 | 142 | 67.6 | | 3 | 50 | 53 | 5.7 |
| ≥50 | 1 | 53 | 52 | 105 | 50.5 | 0 | 1 | 70 | 71 | 1.4 |
| UD 1-12 | | | | | | | | | | |
| Any | 9 | 77 | 230 | 307 | 25.1 | 1 | 1 | 155 | 156 | 0.6 |
| <20 | 2 | 29 | 33 | 62 | 46.8 | | 1 | 32 | 33 | 3 |
| 20-<50 | 5 | 36 | 104 | 140 | 25.7 | | 0 | 53 | 53 | 0 |
| ≥50 | 1 | 12 | 93 | 105 | 11.4 | 1 | 0 | 70 | 70 | 0 |
| UD 1-18 | | | | | | | | | | |
| Any | 33 | 24 | 258 | 282 | 8.5 | 16 | 0 | 141 | 141 | 0 |
| <20 | 5 | 8 | 51 | 59 | 13.6 | 5 | 0 | 28 | 28 | 0 |
| 20-<50 | 20 | 12 | 113 | 125 | 9.6 | 7 | 0 | 46 | 46 | 0 |
| ≥50 | 8 | 4 | 94 | 98 | 4.1 | 4 | 0 | 67 | 67 | 0 |
| | | | | | | | | | | |
| **Lofa** | | | | | | | | | | |
| UD 1-6 | | | | | | | | | | |
| Any | 4 | 173 | 23 | 196 | 88.3 | 0 | 8 | 91 | 99 | 8.1 |
| <20 | 3 | 76 | 4 | 80 | 95 | | 6 | 36 | 42 | 14.3 |
| 20-<50 | 1 | 73 | 10 | 83 | 88 | | 2 | 37 | 39 | 5.1 |
| ≥50 | 0 | 24 | 9 | 33 | 72.7 | 0 | 0 | 18 | 18 | 0 |

| Area | Moxidectin | | | | | Ivermectin | | | | |
|---|---|---|---|---|---|---|---|---|---|---|
| **IoI** | **ND** | **n UD Yes** | **n UD No** | **N** | **% with UD** | **ND** | **n UD Yes** | **n UD No** | **N** | **% with UD** |
| UD 1-12 | | | | | | | | | | |
| Any | 13 | 99 | 88 | 187 | 52.9 | 6 | 1 | 92 | 93 | 1.1 |
| <20 | 6 | 52 | 25 | 77 | 67.5 | 1 | 0 | 41 | 41 | 0 |
| 20-<50 | 6 | 36 | 42 | 78 | 46.2 | 3 | 1 | 35 | 36 | 2.8 |
| ≥50 | 1 | 11 | 21 | 32 | 34.4 | 2 | 0 | 16 | 16 | 0 |
| UD 1-18 | | | | | | | | | | |
| Any | 20 | 57 | 123 | 180 | 31.7 | 7 | 1 | 91 | 92 | 1.1 |
| <20 | 9 | 31 | 43 | 74 | 41.9 | 2 | 0 | 40 | 40 | 0 |
| 20-<50 | 10 | 23 | 51 | 74 | 31.1 | 3 | 1 | 35 | 36 | 2.8 |
| ≥50 | 1 | 3 | 29 | 32 | 9.4 | 2 | 0 | 16 | 16 | 0 |
| | | | | | | | | | | |
| Nkwanta | | | | | | | | | | |
| UD 1-6 | | | | | | | | | | |
| Any | 3 | 117 | 38 | 155 | 75.5 | 0 | 1 | 82 | 83 | 1.2 |
| <20 | 0 | 33 | 9 | 42 | 78.6 | | 0 | 24 | 24 | 0 |
| 20-<50 | 1 | 62 | 13 | 75 | 82.7 | | 1 | 37 | 38 | 2.6 |
| ≥50 | 2 | 22 | 16 | 38 | 57.9 | 0 | 0 | 21 | 21 | 0 |
| UD 1-12 | | | | | | | | | | |
| Any | 5 | 39 | 114 | 153 | 25.5 | 1 | 1 | 81 | 82 | 1.2 |
| <20 | 1 | 18 | 23 | 41 | 43.9 | 1 | 0 | 23 | 23 | 0 |
| 20-<50 | 2 | 17 | 57 | 74 | 23 | | 1 | 37 | 38 | 2.6 |
| ≥50 | 2 | 4 | 34 | 38 | 10.5 | 0 | 0 | 21 | 21 | 0 |
| UD 1-18 | | | | | | | | | | |
| Any | 63 | 10 | 85 | 95 | 10.5 | 33 | 0 | 50 | 50 | 0 |
| <20 | 10 | 6 | 26 | 32 | 18.8 | 9 | 0 | 15 | 15 | 0 |
| 20-<50 | 29 | 1 | 46 | 47 | 2.1 | 15 | 0 | 23 | 23 | 0 |
| ≥50 | 24 | 3 | 13 | 16 | 18.8 | 9 | 0 | 12 | 12 | 0 |

IoI: IoI category; N: Number of participants included in the analysis; NC: OR not calculated since Odds for ivermectin group 0; ND: number of participants with a missing data point for at least one of the relevant time points and excluded from the analysis; n UD No: number of participants with data points at all relevant timepoints and detectable SmfD included in the analysis; n UD yes: number of participants with data points at all relevant timepoints and undetectable SmfD included in the analysis, OR: Odds ratio; UD 1-6, UD 1-12, UD 1-18: undetectable SmfD at M1 and M6, at M1, M6 and M12, at M1, M6, M12, M18, respectively.

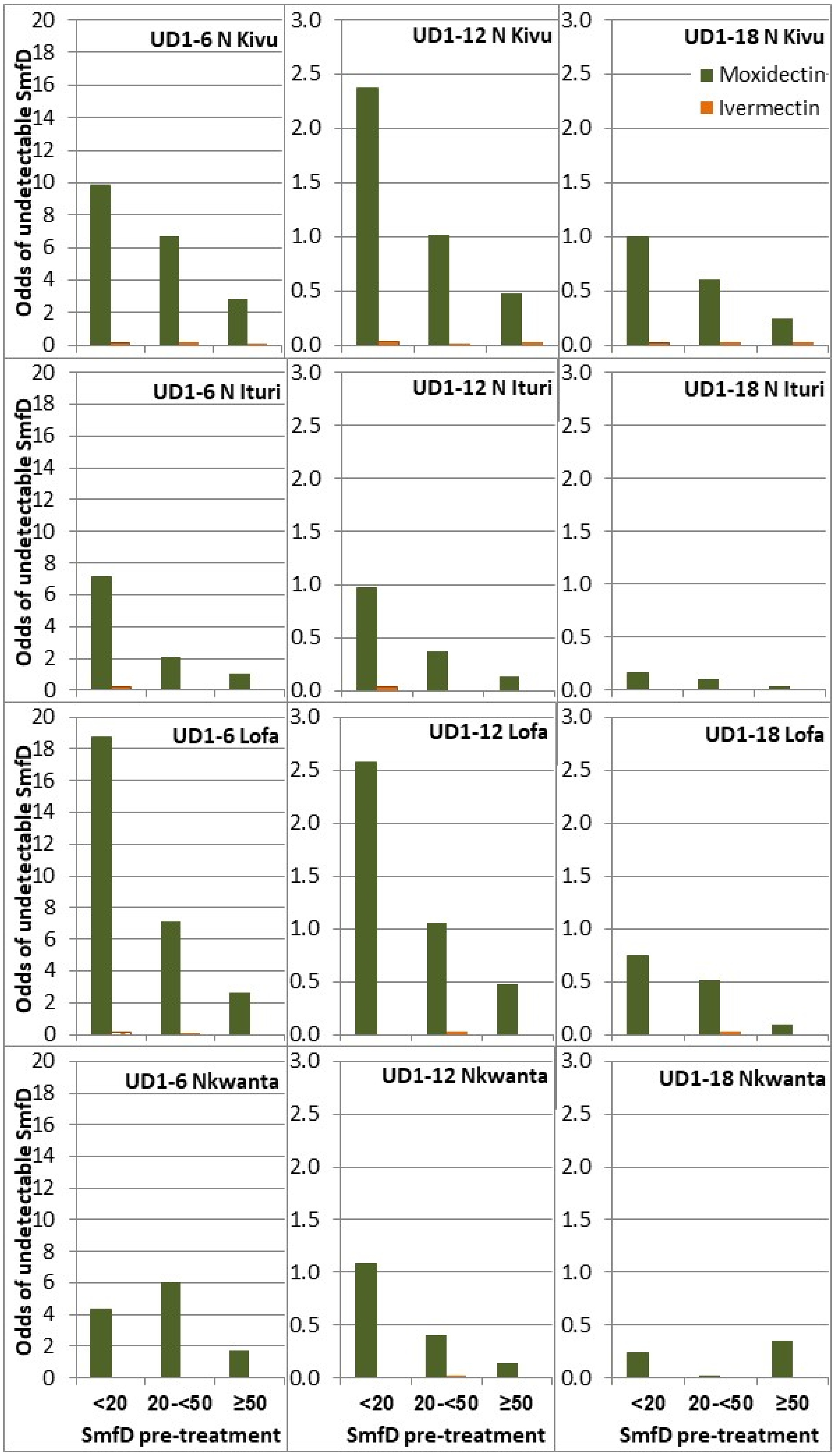


**Fig S4: Odds for UD from 1 to 6, 12 or 18 months by IoI and study area**

SmfD: skin microfilariae density, UD1-6, UD 1-12, UD 1-18 undetectable levels of SmfD at month 1 and 6, at months 1, 6 and 12, and at month 1, 6, 12 and 18, respectively (for number with follow up data at each relevant time point, see Table S11).

**Table S12 Logistic model derived odds for undetectable levels of skin microfilariae from month 1 sustained to month 6, month 12 or month 18 among participants treated with moxidectin or ivermectin by IoI across and by study area**

| Study area | Moxidectin: | | | | | | | | | Ivermectin | | | | | | | | |
|---|---|---|---|---|---|---|---|---|---|---|---|---|---|---|---|---|---|---|
| IoI | M1 and M6 | | | M1, M6, and M12 | | | M1, M6, M12, and M18 | | | M1 and M6 | | | M1, M6, and M12 | | | M1, M6, M12, and M18 | | |
| | Odds | LL | UL | Odds | LL | UL | Odds | LL | UL | Odds | LL | UL | Odds | LL | UL | Odds | LL | UL |
| **Across all** | | | | | | | | | | | | | | | | | | |
| 10-<20 | 8.48 | 5.12 | 14.05 | 1.54 | 0.92 | 2.59 | 0.41 | 0.18 | 0.93 | 0.13 | 0.07 | 0.23 | 0.02 | 0.00 | 0.05 | 0.01 | 0.00 | 0.04 |
| 20-<50 | 4.33 | 2.88 | 6.51 | 0.62 | 0.38 | 1.02 | 0.24 | 0.11 | 0.54 | 0.08 | 0.04 | 0.16 | 0.02 | 0.00 | 0.05 | 0.01 | 0.00 | 0.06 |
| 50-<80 | 2.59 | 1.60 | 4.20 | 0.27 | 0.15 | 0.50 | 0.10 | 0.04 | 0.27 | 0.03 | 0.01 | 0.10 | 0.01 | 0.00 | 0.07 | 0.01 | 0.00 | 0.08 |
| ≥80 | 0.90 | 0.52 | 1.58 | 0.23 | 0.11 | 0.48 | 0.09 | 0.03 | 0.32 | 0.00 | 0.00 | Infty | 0.00 | 0.00 | Infty | 0.00 | 0.00 | Infty |
| **Nord-Kivu** | | | | | | | | | | | | | | | | | | |
| 10-<20 | 9.84 | 4.85 | 19.97 | 2.37 | 1.48 | 3.79 | 1.00 | 0.60 | 1.66 | 0.15 | 0.07 | 0.34 | 0.04 | 0.01 | 0.16 | 0.03 | 0.00 | 0.21 |
| 20-<50 | 6.70 | 4.11 | 10.90 | 1.02 | 0.72 | 1.45 | 0.60 | 0.39 | 0.94 | 0.18 | 0.09 | 0.40 | 0.02 | 0.00 | 0.14 | 0.03 | 0.00 | 0.22 |
| ≥50 | 2.89 | 1.61 | 5.17 | 0.48 | 0.27 | 0.84 | 0.25 | 0.12 | 0.50 | 0.05 | 0.01 | 0.23 | 0.03 | 0.00 | 0.20 | 0.04 | 0.00 | 0.26 |
| **Nord Ituri** | | | | | | | | | | | | | | | | | | |
| 10-<20 | 7.11 | 3.37 | 15.01 | 0.96 | 0.58 | 1.61 | 0.17 | 0.08 | 0.34 | 0.18 | 0.07 | 0.47 | 0.03 | 0.00 | 0.24 | 0.00 | 0.00 | Infty |
| 20-<50 | 2.12 | 1.48 | 3.04 | 0.37 | 0.26 | 0.52 | 0.11 | 0.06 | 0.20 | 0.06 | 0.02 | 0.20 | 0.00 | 0.00 | Infty | 0.00 | 0.00 | Infty |
| ≥50 | 1.03 | 0.70 | 1.51 | 0.13 | 0.07 | 0.24 | 0.04 | 0.02 | 0.12 | 0.01 | 0.00 | 0.10 | 0.00 | 0.00 | Infty | 0.00 | 0.00 | Infty |
| **Lofa** | | | | | | | | | | | | | | | | | | |
| 10-<20 | 18.71 | 6.81 | 51.41 | 2.57 | 1.81 | 3.66 | 0.75 | 0.46 | 1.22 | 0.16 | 0.07 | 0.39 | 0.00 | 0.00 | Infty | 0.00 | 0.00 | Infty |
| 20-<50 | 7.09 | 3.63 | 13.83 | 1.06 | 0.65 | 1.72 | 0.52 | 0.31 | 0.87 | 0.05 | 0.01 | 0.22 | 0.03 | 0.00 | 0.22 | 0.03 | 0.00 | 0.23 |
| ≥50 | 2.67 | 1.23 | 5.76 | 0.48 | 0.22 | 1.05 | 0.09 | 0.03 | 0.30 | 0.00 | 0.00 | Infty | 0.00 | 0.00 | Infty | 0.00 | 0.00 | Infty |
| **Nkwanta** | | | | | | | | | | | | | | | | | | |
| 10-<20 | 4.32 | 1.92 | 9.73 | 1.08 | 0.61 | 1.90 | 0.24 | 0.09 | 0.64 | 0.00 | 0.00 | Infty | 0.00 | 0.00 | Infty | 0.00 | 0.00 | Infty |
| 20-<50 | 6.04 | 3.06 | 11.92 | 0.40 | 0.22 | 0.74 | 0.03 | 0.00 | 0.21 | 0.03 | 0.00 | 0.23 | 0.03 | 0.00 | 0.21 | 0.00 | 0.00 | Infty |
| ≥50 | 1.73 | 0.85 | 3.54 | 0.14 | 0.05 | 0.43 | 0.35 | 0.09 | 1.35 | 0.00 | 0.00 | Infty | 0.00 | 0.00 | Infty | 0.00 | 0.00 | Infty |

Infty – infinity, due to number of participants with undetectable SmfD = 0 (see Table S11), LL lower limit, UL upper limit of the 95% confidence interval
Odds and 95% confidence intervals obtained from logistic model with treatment, IoI category and IoI category*treatment interaction as fixed effects. For the across study area analysis, the study area was a random effect. Only individuals who had SmfD determined at each of the relevant time points were included in these analyses.

**Table S13 Logistic model derived odds ratios for undetectable levels of skin microfilariae from month 1 sustained to month 6, month 12 or month 18 among participants treated with moxidectin relative to among ivermectin treated participants by IoI by study area**

| Study Area | p | Odds Ratio | LL OR | UL OR | p | Odds Ratio | LL OR | UL OR | p | Odds Ratio | LL OR | UL OR |
|---|---|---|---|---|---|---|---|---|---|---|---|---|
| **IOI (mf/mg)** | **M1 and M6** | | | | **M1, M6 and M12** | | | | **M1, M6, M12 and M18** | | | |
| **Across** | | | | | | | | | | | | |
| 10-<20 | <.0001 | 67.461 | 35.771 | 127.224 | <.0001 | 98.272 | 29.771 | 324.389 | <.0001 | 76.591 | 10.355 | 566.524 |
| 20-<50 | <.0001 | 51.528 | 28.196 | 94.165 | <.0001 | 39.740 | 12.275 | 128.659 | <.0001 | 20.990 | 4.994 | 88.225 |
| 50-<80 | <.0001 | 87.156 | 26.074 | 291.329 | 0.0011 | 29.529 | 3.900 | 223.570 | 0.0245 | 10.652 | 1.356 | 83.684 |
| ≥80 | | >999.999 | <0.001 | >999.999 | | >999.999 | <0.001 | >999.999 | | >999.999 | <0.001 | >999.999 |
| **Nord Kivu** | | | | | | | | | | | | |
| 10-<20 | <.0001 | 65.173 | 21.956 | 193.455 | <.0001 | 62.792 | 13.93 | 283.052 | 0.0008 | 35.038 | 4.452 | 275.777 |
| 20-<50 | <.0001 | 36.366 | 14.461 | 91.456 | 0.0001 | 52.91 | 6.983 | 400.877 | 0.0041 | 20.38 | 2.627 | 158.094 |
| ≥50 | <.0001 | 53.16 | 11.471 | 246.35 | 0.0071 | 17.321 | 2.182 | 137.495 | 0.072 | 6.99 | 0.84 | 58.185 |
| **Nord Ituri** | | | | | | | | | | | | |
| 10-<20 | <.0001 | 39.218 | 11.703 | 131.423 | 0.0014 | 29.02 | 3.691 | 228.18 | | >999.999 | <0.001 | >999.999 |
| 20-<50 | <.0001 | 34.911 | 10.302 | 118.303 | | >999.999 | <0.001 | >999.999 | | >999.999 | <0.001 | >999.999 |
| ≥50 | <.0001 | 71.111 | 9.481 | 533.344 | | >999.999 | <0.001 | >999.999 | | >999.999 | <0.001 | >999.999 |
| **Lofa** | | | | | | | | | | | | |
| 10-<20 | <.0001 | 115.007 | 30.296 | 436.59 | | >999.999 | <0.001 | >999.999 | | >999.999 | <0.001 | >999.999 |
| 20-<50 | <.0001 | 137.692 | 28.387 | 667.873 | 0.0008 | 35.537 | 4.448 | 283.957 | 0.0078 | 16.833 | 2.114 | 134.029 |
| ≥50 | | >999.999 | <0.001 | >999.999 | | >999.999 | <0.001 | >999.999 | | >999.999 | <0.001 | >999.999 |
| **Nkwanta** | | | | | | | | | | | | |
| 10-<20 | | >999.999 | <0.001 | >999.999 | | >999.999 | <0.001 | >999.999 | | >999.999 | <0.001 | >999.999 |
| 20-<50 | <.0001 | 196.761 | 23.954 | >999.999 | 0.0139 | 14.546 | 1.732 | 122.176 | | >999.999 | <0.001 | >999.999 |
| ≥50 | | >999.999 | <0.001 | >999.999 | | >999.999 | <0.001 | >999.999 | | >999.999 | <0.001 | >999.999 |

LL OR Lower Limit of 95% Confidence Interval of the Odds Ratio, OR Odds Ratio, UL Upper limit of 95% Confidence Interval of the Odds Ratio,
OR, LL OR and UL OR >999.999 indicate that these values could not be estimated by the model because the number of ivermectin treated participants with undetectable SmfD was 0 (see Table S11).
Odds ratio, 95% Confidence Intervals and p values obtained from logistic model with treatment, IoI category and IoI category*treatment interaction as fixed effects. For the across study area analysis, the study area was a random effect. Only individuals who had SmfD determined at each of the relevant time points were included in these analyses.

# References


1. Ngoumou P, Walsh JF A Manual for Rapid Epidemiological Mapping of Onchocerciasis. 1993.
2. Ngoumou P, Walsh JF, Mace JM A rapid mapping technique for the prevalence and distribution of onchocerciasis: a Cameroon case study. Ann Trop Med Parasitol. 1994;88: 463-474.
3. Coffeng LE, Pion SD, O'Hanlon S, Cousens S, Abiose AO, Fischer PU, Remme JH, Dadzie KY, Murdoch ME, de Vlas SJ, Basanez MG, Stolk WA, Boussinesq M Onchocerciasis: the pre-control association between prevalence of palpable nodules and skin microfilariae. PLoS Negl Trop Dis. 2013;7: e2168.
4. Noma M, Nwoke BE, Nutall I, Tambala PA, Enyong P, Namsenmo A, Remme J, Amazigo UV, Kale OO, Seketeli A Rapid epidemiological mapping of onchocerciasis (REMO): its application by the African Programme for Onchocerciasis Control (APOC). Ann Trop Med Parasitol. 2002;96 Suppl 1: S29-S39.
5. Noma M, Zoure HG, Tekle AH, Enyong PA, Nwoke BE, Remme JH The geographic distribution of Onchocerciasis in the 20 participating countries of the African programme for Onchocerciasis control: (1) priority areas for ivermectin treatment. Parasit Vectors. 2014;7: 325.
6. Fobi G, Yameogo L, Noma M, Aholou Y, Koroma JB, Zoure HM, Ukety T, Lusamba-Dikassa PS, Mwikisa C, Boakye DA, Roungou JB Managing the Fight against Onchocerciasis in Africa: APOC Experience. PLoS Negl Trop Dis. 2015;9: e0003542.
7. Zoure HG, Noma M, Tekle AH, Amazigo UV, Diggle PJ, Giorgi E, Remme JH The geographic distribution of onchocerciasis in the 20 participating countries of the African Programme for Onchocerciasis control: (2) pre-control endemicity levels and estimated number infected. Parasit Vectors. 2014;7: 326.
8. Seketeli A, Adeoye G, Eyamba A, Nnoruka E, Drameh P, Amazigo UV, Noma M, Agboton F, Aholou Y, Kale OO, Dadzie KY The achievements and challenges of the African Programme for Onchocerciasis Control (APOC). Ann Trop Med Parasitol. 2002;96 Suppl 1: S15-S28.
9. Prost A, Hervouet JP, Thylefors B Epidemiologic status of onchocerciasis. Bull World Health Organ. 1979;57: 655-662.
10. Remme J, Ba O, Dadzie KY, Karam M A force-of-infection model for onchocerciasis and its applications in the epidemiological evaluation of the Onchocerciasis Control Programme in the Volta River basin area. Bull World Health Organ. 1986;64: 667-681.
