# Supplementary material for: Effect of a single dose of 8 mg moxidectin or 150 μg/kg ivermectin on O. volvulus skin microfilariae in a randomized trial: Differences between areas in the Democratic Republic of the Congo, Liberia and Ghana and impact of intensity of infection
Source: PLoS Negl Trop Dis. 2022 Apr 27;16(4):e0010079. doi: 10.1371/journal.pntd.0010079 (PMC9084535; doi:10.1371/journal.pntd.0010079)
Supplement: Attachment — Submitted filename: Review Bakajika et al Moxidectin 2022.pdf [file pntd.0010079.s002.pdf]

This paper by Bakajika et al, summarize data already published with the huge effect of Moxidectin on onchocerciasis compared to ivermectin after the phase 3 trials across the study sites in DRC, Liberia and Ghana. The main message of the present publication is that the difference between Moxidectin and ivermectin differs according to the study areas. Also, the suboptimal response to ivermectin was described in naïve ivermectin areas. All these information are known from previous data and previous publications. Nevertheless, this paper highlights the priority in the use of Moxidectin and insists on the adding value of this molecule for the elimination of onchocerciasis. Considering the priority and the emergency to eliminate onchocerciasis in Sub-Saharan Africa, this new tool (Moxidectin) should be popularized and widely used for the benefit of the affected populations.

Minor comment

SOMR: it is not clear whether this Sub Optimal Microfilariae Response on Sub Optimal Microfilariae Responders (Line 459, page 30 and Table 6 and 7).

OMR: Optimal Microfilariae Response is used in Table 7, but nowhere in the text.
