# Supplementary material for: Effect of a single dose of 8 mg moxidectin or 150 μg/kg ivermectin on O. volvulus skin microfilariae in a randomized trial: Differences between areas in the Democratic Republic of the Congo, Liberia and Ghana and impact of intensity of infection
Source: PLoS Negl Trop Dis. 2022 Apr 27;16(4):e0010079. doi: 10.1371/journal.pntd.0010079 (PMC9084535; doi:10.1371/journal.pntd.0010079)
Supplement: Attachment — Submitted filename: PNTD-D-21-01732_Author Response to reviewer comments.pdf [file pntd.0010079.s003.pdf]

Response to Reviewer 1 (Anonymous):

1 Are the objectives of the study clearly articulated with a clear testable hypothesis stated?

Yes, in the manuscript but not in the Abstract nor in the Author summary.

> **Response:** Since this manuscript reports additional analysis of data from a study already published, we do not consider it helpful to use the limited space available in Abstract and Author summary to refer to the objectives and hypothesis of the study. The additional analyses for this publication are clearly described in the Abstract: 'We now analysed efficacy by study area and pre-treatment SmfD (intensity of infection, IoI).

2 Is the study design appropriate to address the stated objectives?

Yes, to a great extent. Line 249, please define or explain what « adjusted » stands for.

> **Response:** The variables adjusted for are included in the model described in the preceding sentence that specifies the analysis from which the adjusted geometric means were derived.
>
> In response to this comment, we have added (obtained from the model) to the sentence.

3 Are the results clearly and completely presented?

Yes, mostly. However, the « treatment differences » as provided in the abstract (line 66) are not apprehensible without a clear definition of what that means. Given the complexity of how this criterium was defined, I would recommend using different statistics to provide meaningful results in the abstract.

> **Response**: We have revised the abstract to specify how treatment differences were calculated. Given the word limit for the abstract, we deleted the first sentence of the abstract.

4 Are the conclusions supported by the data presented?

Yes, but they need to be given in the context of the clinical trial: results after a very first dose of moxidectin or ivermectin. The same comparison after second or subsequent treatments would certainly result in much smaller - if any - differences.

> **Response:** We do not understand this comment.
>
> The Abstract and Methods specify that data from a single dose trial in CDTI-naïve areas are analysed and the Discussion and Conclusions do not make any claims relating to repeated treatments with ivermectin or moxidectin with one exception, which is refers to the results of a published modelling study.
>
> Since the reviewer is not specific about the comparison referred to (between areas, between pre-treatment IoI, between drugs), nor about the data from which the reviewer derives the statement 'would certainly result in smaller differences' addressing this comment was not self evident.
>
> To address this comment, we added 'one treatment with …' to the Author Summary and we added to the Discussion information on the currently ongoing, planned and completed studies of moxidectin and the information they will provide for WHO and countries to decide whether to include moxidectin in guidelines and policies.

5 Are the limitations of analysis clearly described?

No, but there is matter to do so (eg: see previous point).

> **Response:** see our response to the previous point.

6 Editorial and Data Presentation Modifications

- Because the paper is quite lengthy. I recommend to move the section stating line 272 and ending line 312 to the supplementary information section. It is only marginally relevant to the subsequent analyses.

Response: We implemented the recommendation of the reviewer.

- Line 233: « for this manuscript » can be omitted

  Response: We implemented the recommendation of the reviewer.

- Line 264: replace « explained » by a more appropriate expression (eg: depending on)

  Response: We replaced 'explained' by 'a function of'

- Line 424: UD is defined for undetectable SmfD at line 424 although the expression has been previously used, and is again used in subsequent lines. Reconsider the appropriate use of UD.

  Response: We have deleted UD in that sentence since UD is indeed already defined in the Methods section.

- Line 515: « typical » should be « atypical »

  Response: We have clarified the sentence by removing quotation marks. We also added the term 'adequate' used in the original publication on suboptimal response to ivermectin.

- Line 538: « a maximum SmfD reduction from pre-treatment by 100% » would better read as « mf clearance » of equivalent expression.

  Response: We agree that 'mf clearance' would read better.

  We have nevertheless retained the expression 'maximum SmfD reduction from pre-treatment by 100%' which is the technically correct expression. 'mf clearance' could be misinterpreted as a claim that there are no microfilariae in the skin anymore. However, that is not a conclusion that can be drawn from a reduction of measured SmfD by 100% (i.e. no microfilariae detected in any of the four skin snips) given the sensitivity of microfilariae detection via skin snips. This is also the reason why we are using the expression 'undetectable' skin microfilariae density.

- Line 638: delete one of the comas

  Response: We implemented the recommendation of the reviewer.

7 Summary and General Comment

- This is a companion paper to an important already published paper that showed an increased efficacy (and similar safety profile) of a single oral treatment of moxidectin over a single oral dose of ivermectin on Onchocerca volvulus microfilaridermia. The present paper had the specific objective of comparing the efficacy of the two drug across four different study sites.

  Response: Comparing the efficacy of the two drugs across the four study sites was not the objective of the present paper but of the already published paper the reviewer mentions in the comment (Opoku et al. 2018).

  We have examined the text in the introduction that summarizes our objectives to see whether clarification is needed: 'Here, we are reporting analyses conducted to investigate whether the efficacy of the drugs differs between study areas, to better characterize the dependency of the treatment difference on pre-treatment SmfD and to explore the significant inter-individual variability of response to ivermectin the raw data suggested. Furthermore, we are reporting the *O. volvulus* infection relevant data obtained in all individuals screened by study area'.

  We find that the text is very clear.

- The main conclusion of this trial is that moxidectin seems to be more beneficial when skin mf density is higher.

  Response: A relatively higher benefit of moxidectin compared to ivermectin for individuals with higher pre-treatment skin mf densities is only ONE conclusion.

  Equally, and arguably even more important, conclusions relate to the objectives 'to investigate whether the efficacy of the drugs differs between study areas' and 'to explore the significant inter-individual variability of response to ivermectin the raw data suggested'. This is reflected in the Abstract 'Conclusions/Significance' as well as in the fact that the

majority of the Discussion does not deal with the relative benefit of moxidectin and ivermectin in dependence of pre-treatment skin mf density.

We acknowledge that the number of objectives and analyses (not counting the analysis of the screening data) and consequently large number of results to be discussed, have lead to the main conclusions not being well expressed in the 'Discussion and Conclusions'.

To address this, we revised the manuscript to add a 'Conclusions' section.

- I think that the authors'enthusiasm regarding the promising results obtained with moxidectin may guide them to promote moxidectin over ivermectin beyond avaiabe evidence. Indeed, the authors should keep in mind that their results were obtained as part of a clinical trial in which all participants were treated for the first time. Moxidectin, likewise ivermectin, is to be used repeatedly (annually, semi-annually, or possibly at longer intervals if the effects are long standing). The conclusion obtained in this paper may not stand after a second treatment given six months or one year later.

  Response: We have carefully examined the manuscript and in particular 'Discussion and Conclusions' for phrasing that could be considered as claims relating to the relative effect of repeated treatments, i.e. language promoting moxidectin over ivermectin beyond available evidence that the reviewer might be referring to.

  We find that we have been appropriately conservative in phrasing the sentences about the potential value of moxidectin for onchocerciasis control and elimination. Specifically the final paragraph in the original manuscript states 'that moxidectin treatment **may** be particularly advantageous for achieving elimination of *O. volvulus* transmission in areas where infection prevalence remains high despite long-term CDTI, where transmission seasons are long or have two peaks, where elimination programmes face operational barriers to implementing at least annual CDTI and where SOR to ivermectin has been observed.

  The only time that the relative effect of repeat treatment with moxidectin compared to ivermectin is invoked involves the results of a published modelling study with reference (Turner et al) that we included in support of the 'may' statement above.

- Both inter-individual heterogeneity and treatment difference will certainly be reduced during round 2, 3 and so on, compared to round 1 of treatment. This should be addressed in the discussion.

  Response: Unfortunately, the reviewer has not specified on which evidence the reviewer bases this certainty. We are not aware of any publications that have analysed inter-individual heterogeneity in response to successive ivermectin treatments. A study that will provide data on inter-individual heterogeneity and treatment differences after repeat treatments with moxidectin or ivermectin is only ongoing.

  To address this and the previous comment, we have added a paragraph at the end of the Discussion which

  a) includes information on all ongoing, completed and planned studies of moxidectin and the additional data they will provide to inform WHO and country decisions on whether to include moxidectin in onchocerciasis elimination guidelines and policies,

  b) clarifies that WHO and countries base their decisions on inclusion of new treatment options in guidelines and policies on systematic reviews and evaluations of the data, not on 'promotion' by investigators/authors in their publications.

- In addition, the authors decided to use sub-optimal response criteria defined for ivermectin to assess possible sub-optimal response with moxidectin. Firstly, relevance of those criteria when not associated with genetic data is arguable, and secondly, other criteria may need to be defined for moxidectin. This should at last be addressed in the discussion. In addition, see my point above on the expected decreasing difference in treatment efficacy after several treatments.

Response: The reviewer appears to suggest that the efficacy of different drugs for the same indication should be evaluated based on different criteria. This is a concept that we are not familiar with and do not consider appropriate.

The reviewer's comment suggests that it was not clear to this reviewer that the suboptimal response criteria established by others were one of a number of measures we used to describe the inter-individual variability in post-treatment SmfD changes.

To address this comment, we have added:

(1) to the Methods the list of measures used to describe the inter-individual variability in post-treatment SmfD changes,

(2) to the Discussion the context and origin of the suboptimal response criteria to clarify the significance of the results of these analyses. (Note: to avoid repetition, we adjusted original text further below.

- In addition, for the time being, moxidectin is not recommended for use in children, therefore « CDTM » is not an option for now. I would recommend the authors to temper their conclusions or perspective regarding the long term effect of moxidectin. Maybe it will be much more efficient that ivermectin (allowing for reducing the duration of intervention for similar coverage/compliance figures), maybe the « treatment difference » will only be significant after the initial treatment.

Response: We have carefully examined the manuscript for any 'conclusions or perspectives regarding the long-term effect of moxidectin' that the reviewer is referring to.

The only reference to long-term effect of moxidectin is via a published modelling study (Turner et al.).

As noted above, we have been appropriately conservative with any statements regarding future use of moxidectin through choice of the words 'provided', 'may', 'would', or 'could', notably:

'**Provided** WHO and countries include moxidectin in guidelines and policies, this information **could** help decisions on when and where to use moxidectin.'

'… the Phase 2 and Phase 3 study safety data suggest that moxidectin **may** have the safety profile required for Community-Directed Treatment with Moxidectin (CDTM)' (Line XXX original manuscript, Line 154 ff revised manuscript)

'To include a population as similar to that which **would** be participating in CDTM as possible …'.

'… that moxidectin treatment **may** be particularly advantageous for achieving elimination of *O. volvulus* transmission in areas where infection prevalence remains high despite long-term CDTI, where transmission seasons are long or have two peaks, where elimination programmes face operational barriers to implementing at least annual CDTI and where SOR to ivermectin has been observed.'

- Line 86: « Ivermectin may not be sufficiently efficacious to achieve elimination everywhere » is purely speculative. Persistent onchocerciasis is mostly due to a mis-use of ivermectin (low therapeutic coverage, hectic compliance…) not to a lack of efficacy when it is actually used. Onchocerciasis has been eliminated using ivermectin only. Use of moxidectin for community treatment will

Response: This statement in the original abstract is consistent with current literature and conclusions of expert consultations, one of which we reference in the introduction. (the sentence is deleted in the revised manuscript to make space for information on how treatment differences were calculated, see above)

Our manuscript is not a review of progress and prospects towards elimination with CDTI, hence we included some, but not all references on this topic, as well as one, not all possible references to expert consultations, in the Introduction.

To address this comment we have added text and references for ongoing research into alternative treatment strategies.

The fact that increasing therapeutic coverage will increase CDTI effectiveness is well recognized as are the challenges countries face for achieving and maintaining or increasing high therapeutic coverage and addressing systematic non-compliance.

Given these challenges, we find the reviewer's characterization of low therapeutic coverage as 'mis-use of ivermectin' troublesome. We are not familiar with the concept of 'hectic compliance' and thus cannot respond to that.

Elimination, which can only be claimed once WHO has certified it, with ivermectin alone has been achieved to date in the small endemic areas in the Americas which were either hypo- or meso-endemic or included only small populations in hyperendemic areas.

To address this comment we have added a reference to the Introduction which provides more details on the number of hypo-, meso- or hyperendemic communities and population size in the Americas than the reference we had already provided.

- Line 558: « long term » may be omitted from the sentence

  Response: we deleted 'long term'

- Lines 561-562: IoI reflects the number of reproductive adults but also the immunological tolerance of the host towards microfilariae.

  Response: we added this information.

Response to Reviewer 2 (Prof. Joseph Kamgno):

8 Editorial and Data Presentation Modifications

- SOMR: it is not clear whether this Sub Optimal Microfilariae Response on Sub Optimal Microfilariae Responders (Line 459, page 30 and Table 6 and 7).

  Response: We have deleted 'SOMR' in that sentence and adjusted the subsequent sentence so that SOMR refers throughout to suboptimal microfilariae response, not responders.

  We also reviewed the use of SOR throughout the manuscript and adjusted where necessary the grammar to ensure that SOR refers to suboptimal response not suboptimal responders.

- OMR: Optimal Microfilariae Response is used in Table 7, but nowhere in the text.

  Response: We use this abbreviation only in the table with an explanation in the legend since 'Not SOMR80' would increase the width of the relevant columns unnecessarily.

- This paper by Bakajika et al, summarize data already published with the huge effect of Moxidectin on onchocerciasis compared to ivermectin after the phase 3 trials across the study sites in DRC, Liberia and Ghana. The main message of the present publication is that the difference between Moxidectin and ivermectin differs according to the study areas. Also, the suboptimal response to ivermectin was described in naïve ivermectin areas. All these information are known from previous data and previous publications. Nevertheless, this paper highlights the priority in the use of Moxidectin and insists on the adding value of this molecule for the elimination of onchocerciasis. Considering the priority and the emergency to eliminate onchocerciasis in Sub-Saharan Africa, this new tool (Moxidectin) should be popularized and widely used for the benefit of the affected populations.

  Response: No response required.
